# pH-Responsive Theranostic Colloidosome Drug Carriers Enable Real-Time Imaging of Targeted Thrombolytic Process with Near-Infrared-II for Deep Venous Thrombosis

**DOI:** 10.34133/research.0388

**Published:** 2024-05-29

**Authors:** Yaxin Ye, Zhechang Chen, Shengzhang Zhang, Paul Slezak, Fei Lu, Ruiqi Xie, Dongwon Lee, Guangqian Lan, Enling Hu

**Affiliations:** ^1^ State Key Laboratory of Resource Insects, College of Sericulture, Textile and Biomass Sciences, Southwest University, Chongqing 400715, China.; ^2^ Department of Cardiovascular Medicine, Yueqing People's Hospital, Wenzhou 325699, China.; ^3^ Ludwig Boltzmann Institute for Traumatology, AUVA Research Center, 1200 Vienna, Austria.; ^4^ Department of Bionanotechnology and Bioconvergence Engineering and Department of Polymer·Nano Science and Technology, Jeonbuk National University, Jeonju, Chonbuk 54896, Republic of Korea.; ^5^ School of Fashion and Textiles, The Hong Kong Polytechnic University, Hong Kong.

## Abstract

Thrombosis can cause life-threatening disorders. Unfortunately, current therapeutic methods for thrombosis using injecting thrombolytic medicines systemically resulted in unexpected bleeding complications. Moreover, the absence of practical imaging tools for thrombi raised dangers of undertreatment and overtreatment. This study develops a theranostic drug carrier, Pkr(IR-Ca/Pda-uPA)-cRGD, that enables real-time monitoring of the targeted thrombolytic process of deep vein thrombosis (DVT). Pkr(IR-Ca/Pda-uPA)-cRGD, which is prepared from a Pickering-emulsion-like system, encapsulates both near-infrared-II (NIR-II) contrast agent (IR-1048 dye, loading capacity: 28%) and urokinase plasminogen activators (uPAs, encapsulation efficiency: 89%), pioneering the loading of multiple drugs with contrasting hydrophilicity into one single-drug carrier. Upon intravenous injection, Pkr(IR-Ca/Pda-uPA)-cRGD considerably targets to thrombi selectively (targeting rate: 91%) and disintegrates in response to acidic thrombi to release IR-1048 dye and uPA for imaging and thrombolysis, respectively. Investigations indicate that Pkr(IR-Ca/Pda-uPA)-cRGD enabled real-time visualization of targeted thrombolysis using NIR-II imaging in DVT models, in which thrombi were eliminated (120 min after drug injection) without bleeding complications. This may be the first study using convenient NIR-II imaging for real-time visualization of targeted thrombolysis. It represents the precision medicine that enables rapid response to acquire instantaneous medical images and make necessary real-time adjustments to diagnostic and therapeutic protocols during treatment.

## Introduction

Thrombosis is a frequent cardiovascular condition that is caused by the unexpected activation of platelets and can lead to major health problems [1,2]. Due to their short half-lives, intravenous infusion-administered medicines in conventional thrombolytic therapy rarely target or penetrate thrombus. The potential overdose of a thrombolytic medication may also pose dangers of unintentional bleeding [3–6]. Consequently, precise thrombus targeting is essential for thrombolysis in order to improve the efficacy of medication delivery and decrease overdose. Numerous attempts had been undertaken to create acrobatic drug carriers with the potential to target thrombotic lesions. Previous attempts had been made to guide magnetically responsive drug carriers [7,8] and ultrasound-responsive micromotors [9] to thrombus sites selectively for site-specific thrombolysis. Although these targeting strategies were promising, an alternative targeting mechanism, which relies on intrinsic connections between functional proteins from drug carriers and lesion, shall be attractive as well.

Inspired by targeted cancer therapy, targeted drug delivery for thrombotic diseases is a promising strategy. To achieve this, the drug carrier must contain targeting ligands that recognize and bind to biomarkers or receptors on thrombi, ensuring selective adherence of drug carriers to thrombi. By reviewing proteins having inherent targeting interactions in targeted tumor therapies [10], GPIIb-IIIa (α_IIb_β_3_) integrins are a set of platelet surface proteins that play a critical role in platelet aggregation and are strongly associated with thrombus formation [11,12]. GPIIb-IIIa integrins are normally inactive; however, when activated during thrombus formation, the arginine-glycine-aspartic acid (RGD) motif can selectively bind GPIIb-IIIa integrins from platelets to facilitate platelet aggregation [13,14]. Thus, GPIIb-IIIa integrins and RGD motif are 2 proteins that are mutually exclusive matches. In light of this, it is possible to accomplish selective binding to activated platelets (APs) from thrombi by decorating the drug carrier with cyclic RGD (cRGD) peptides that imitate the RGD pattern [15].

When drug carriers are guided by targeting ligands, responsive release of drugs is the key to effective thrombosis. Some research primarily focused on releasing drugs by ultrasonic excitation [16] and photosensitization [17]. However, these measures had led to over reliance on equipment, resulting in inconvenience. Consequently, different ways for intelligent controlled release had been developed. For example, depending on the varied physiological conditions of the thrombotic tissues or the nature of thrombus substrate, physiological-responsive release had been used as an innovative and intelligent strategy that does not rely on exogenous triggers. Specifically, responsive drug release had be designed to utilize endogenous physiological factors, such as pH [18,19], reactive oxygen species [20], hydrogen peroxide (H_2_O_2_) [21], thrombin [22], and coagulation factors [23], to enable tailored responses to the specific microenvironment of the thrombi. These attempts give considerable evidences that responsive drug release is technically feasible. Among these strategies, the incorporation of pH responsiveness into drug carriers has gained substantial interest [24–26]. This is because thrombus formation sites are often characterized by local tissue hypoxia and accumulation of metabolites, leading to an acidic environment [27,28]. Therefore, pH-responsive systems can take advantage of this intrinsic property to achieve responsive release of drugs.

In deep vein thrombosis (DVT), the anatomical characteristics of deep veins, such as tortuous routes, valves, and constriction, provide substantial challenges for drug delivery and thrombotic imaging [29]. Therefore, diagnostic imaging for DVT is more important than that for other types of thrombosis. Although existing imaging measures include ultrasound [30], photoacoustic imaging [31], computed tomography [28], and magnetic resonance imaging [32], further extending the diversity of imaging approaches is also of interest, especially when coupled with specialized contrast agents. Fluorescence imaging (FLI) in the second near-infrared window (NIR-II, 1,000 to 1,700 nm) is favorable since it requires less specialist equipment and is easier to operate than other imaging techniques. NIR-II FLI appears to be more suited for DVT than the standard first near-infrared window (NIR-I, 700 to 900 nm) FLI due to its increased penetration, greater spatial resolution, and reduced damage to biological components [33–35]. As NIR-II imaging relies heavily on the use of NIR-II- responsive dyes, the selective aggregation of NIR-II-responsive dyes onto thrombus sites is essential for the monitoring of thrombi using NIR-II imaging. Accordingly, both thrombolytic medicines and contrast agents should be placed onto one single-drug carrier. However, there tends to be a paucity of publications that utilized NIR-II dyes for the diagnosis of thrombotic disease [36]. One concern with NIR-II dyes is their extreme hydrophobicity and insolubility in water [37], which impedes their efficient encapsulation into drug carriers for targeted delivery to thrombotic lesion sites. Therefore, rational encapsulation of NIR II dyes in drug carriers is essential and deserves extensive research.

**Fig. 1.**
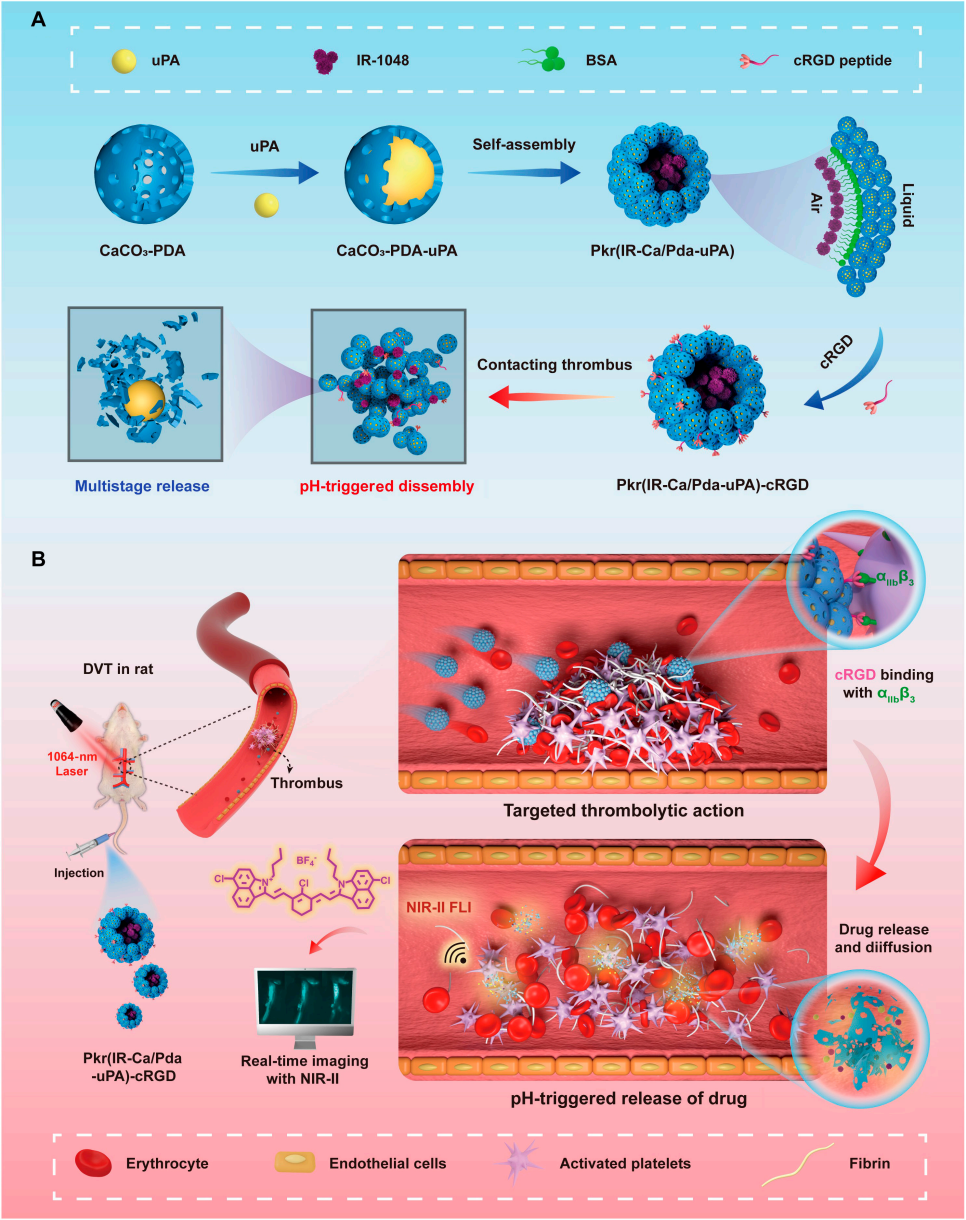
Schematic and theranostic mechanism of Pkr(IR-Ca/Pda-uPA)-cRGD. (A) Preparation and pH-responsive disintegration. (B) Targeted thrombolysis in a rat venous thrombosis model.

In this study, a hierarchical porous colloidosome microsphere, Pkr(IR-Ca/Pda-uPA)-cRGD, has been designed for loading with both thrombolytic medicines and contrast agents for theranostic of thrombus. The strategy combines methods for real-time imaging of thrombi (diagnosis) and thrombi elimination (therapy), as opposed to the independent diagnosis and treatment of thrombosis (Fig. 1A). It requires the drug carrier to possess dual functions in thrombotic imaging and thrombolysis. To begin with, a hollow and porous calcium carbonate-dopamine nanoparticle (CaCO_3_-PDA) serves as the base carrier unit for loading the thrombolytic medication, urokinase plasminogen activators (uPAs), to create the drug-loaded nanocarrier (CaCO_3_-PDA-uPA). Next, CaCO_3_-PDA-uPA further self-assembles in an aqueous dispersion containing the hydrophobic NIR-II contrast agent (IR-1048 dye) to form a Pickering-emulsion-like colloidosome microsphere [Pkr(IR-Ca/Pad-uPA)]. Finally, Pkr(IR-Ca/Pda-uPA) is decorated with cRGD peptide to obtain the final theranostic drug carrier [Pkr(IR-Ca/Pda-uPA)-cRGD], offering the targeting feature for precise drug delivery for DVT. When administered into the blood circulation system, cRGD peptides on Pkr(IR-Ca/Pda-uPA)-cRGD could bond to AP from thrombi to fulfill precise targeting to thrombi; then, the acidic microenvironment of the thrombi would trigger pH-responsive disintegration of Pkr(IR-Ca/Pda-uPA)-cRGD to release both uPA and IR-1048 dye, which are responsible for thrombolysis and thrombotic imaging, respectively (Fig. 1B).

This intelligent thrombolytic drug carrier provides an innovative theranostic strategy for DVT. The colloidosome microspheres are capable of loading both hydrophilic and hydrophobic components easily, thereby breaking through the technical barrier of loading components with varying hydrophilicities in existing drug carriers. Thanks to the encapsulation of both hydrophilic uPA and hydrophobic IR-1048 dye, the dynamic evolution of thrombi of deep veins could be visualized by NIR-II imaging conveniently during treatment, providing more real-time information of in-progress thrombolysis to avoid the risk of overtreatment and undertreatment. To the knowledge of the authors, this may the first study to use the NIR-II imaging for visualization of the targeted thrombolytic process in real time easily. The strategy enables rapid response to unforeseen circumstances during thrombolytic treatment. Due to its universal advantages in theranostic of thrombosis, the drug carrier provides new opportunities for the targeted theranostic for a variety of similar therapies, such as pulmonary embolism and atherosclerosis.

## Results and Discussion

### Design and characterization of Pkr(IR-Ca/Pda-uPA)-cRGD

**Fig. 2.**
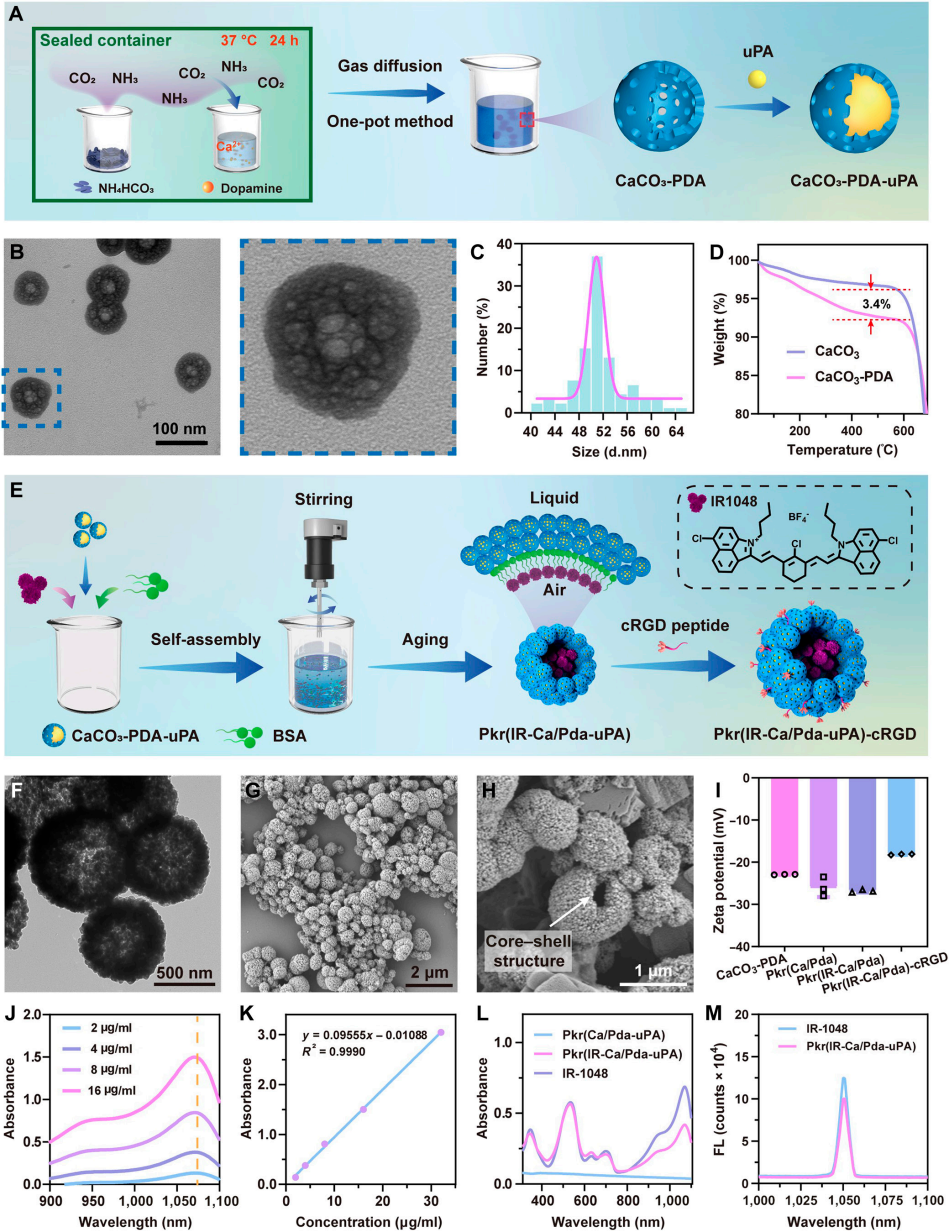
(A) Preparation of CaCO_3_-PDA-uPA. (B) TEM images of CaCO_3_-PDA. (C) Size distribution of CaCO_3_-PDA. (D) Thermogravimetric analysis. (E) Self-assembly of Pkr(IR-Ca/Pda-uPA)-cRGD. (F) TEM images of Pkr(Ca/Pda-uPA). (G) SEM image of Pkr(Ca/Pda-uPA). (H) SEM images of partially broken Pkr(Ca/Pda-uPA). (I) Zeta potential. (J) NIR absorbance spectra of IR-1048. (K) Standard curve of IR-1048. (L) UV-vis-NIR absorbance. (M) Fluorescence spectra. All error bars depicted the mean ± SD of at least 3 independent experiments.

The CaCO_3_-PDA base carrier unit was first constructed for uPA loading (Fig. 2A). It possessed a hollow porous structure (Fig. 2B), high dispersity (Fig. S1A), and a diameter of around 50 nm on average (Fig. 2C). CaCO_3_-PDA contained an equal distribution of N, C, and Ca, as revealed by elemental mapping images (Fig. S1B). The thermogravimetric analysis determined the PDA content to be 3.4% (Fig. 2D), which was essential for the formation of hollow porous structures [38]. Due to the hollow structure of CaCO_3_-PDA, uPA may be loaded into CaCO_3_-PDA with an encapsulation efficiency of up to 89% to create CaCO_3_-PDA-uPA (Fig. S1C), which was hydrophilic, with a static contact angle of approximately 36.4° (Fig. S2).

Thereafter, following self-assembly, CaCO_3_-PDA-uPA encapsulated IR-1048 to form Pkr(IR-Ca/Pda-uPA) (Fig. 2E). During the assembly process, the vigorous stirring of the mixture generated microbubbles by entrapping air; bovine serum albumin (BSA), the biocompatible surfactant with excellent encapsulation capacity, stabilized the microbubbles. At the interface of the microbubbles, a fascinating interaction occurred: the hydrophilic domain of BSA encouraged the self-assembly of CaCO_3_-PDA-uPA, while the hydrophobic domain of BSA bound hydrophobic IR-1048 dye. Pkr(IR-Ca/Pda-uPA) was produced by the self-assembly of hydrophilic CaCO_3_-PDA-uPA and encapsulation of hydrophobic IR-1048 dyes until the microbubble size was sufficiently reduced for tight packing. Figure 2F and G depicts the structure and morphology of Pkr(Ca/Pda-uPA), which was discovered to have an average diameter of around 690 nm and a core–shell structure (Fig. S3A). Figure 2H provides compelling evidence that Pkr(Ca/Pda-uPA) was composed of a vast empty core and an external shell (white arrow). Furthermore, the encapsulation of IR-1048 dye into Pkr(Ca/Pda-uPA) to prepare Pkr(IR-Ca/Pda-uPA) did not impact the structure of Pkr(Ca/Pda-uPA) (Fig. S3B). Cl, the characteristic element of IR-1048 dye, was discovered in elemental mapping (Fig. S3C), indicating the effective encapsulation of IR-1048 dye in Pkr(IR-Ca/Pda-uPA).

Finally, Pkr(IR-Ca/Pda-uPA) was modified by cRGD to produce Pkr(IR-Ca/Pda-uPA)-cRGD, as the amino groups of PDA could react with the carboxyl groups of the cRGD peptide in the presence of 1-(3-dimethylaminopropyl)-3-ethyl carbodiimide hydrochloride (EDC)/N-hydroxysuccinimide (NHS) (Fig. S4). The NMR spectrum shows distinctive absorptions in the range of 6.87 to 8.38 (Fig. S5), which was the evidence that cRGD had been grafted onto the surface of the Pkr(IR-Ca/Pda-uPA)-cRGD [39]. Due to the increased amount of amino groups from grafted cRGD peptides, the zeta potential of Pkr(IR-Ca/Pda-uPA)-cRGD increased to −18.17 mV after cRGD grafting. In addition, it was also important to note that the zeta potential of Pkr(IR-Ca/Pda) was comparable to that of Pkr(Ca/Pda) (Fig. 2I), suggesting the encapsulation of IR-1048 into Pkr(Ca/Pda) did not change the surface charge of Pkr(Ca/Pda) as IR-1048 rarely presented on the surface of Pkr(Ca/Pda).

The NIR absorption of IR-1048 dye demonstrated that a maximum absorbance was found at the wavelength of 1,070 nm (Fig. 2J). According to the standard curve of IR-1048 dye (Fig. 2K), which revealed that a linear relationship between absorption and dye concentration within the range of 2 to 32 μg ml^−1^ was observed, the loading capacity (DL%) of IR-1048 was calculated to be 28.11% (Fig. S6A). Moreover, the apparent absorption peak of Pkr(IR-Ca/Pda-uPA) at 1,070 nm indicated that IR-1048 had been successfully encapsulated by Pkr(IR-Ca/Pda-uPA) (Fig. 2L). As the peak fluorescence absorption of IR-1048 and Pkr(IR-Ca/Pda-uPA) were perfectly aligned at the same wavelength (Fig. 2M), this provided additional evidence that IR-1048 had been encapsulated within Pkr(IR-Ca/Pda-uPA) and confirmed the potential of Pkr(IR-Ca/Pda-uPA) in NIR-II FLI.

During the assembly of CaCO_3_-PDA for preparation of Pkr(IR-Ca/Pda-uPA) and grafting of cRGD onto Pkr(IR-Ca/Pda-uPA) for preparation of Pkr(IR-Ca/Pda-uPA)-cRGD, slight leakages of the loaded uPA in CaCO_3_-PDA were observed in the 2 typical processes, amounting to 5.81% and 12.68%, respectively (Fig. S6B). Fortunately, the net loading percentage of uPA in Pkr(IR-Ca/Pda-uPA)-cRGD remained within an acceptable range at 71%, suggesting that Pkr(IR-Ca/Pda-uPA)-cRGD was reliable and effective for carrying and delivery of uPA for targeted thrombolysis.

### In vitro NIR-II FLI

In vitro imaging was done by evaluating the NIR-II FLI intensity of Pkr(IR-Ca/Pda-uPA)-cRGD at varied concentrations and tissue depths. Imaging intensity was shown to increase with increasing concentration, exhibiting good concentration-dependent properties (Fig. S7A and B). FLI can be fulfilled with a tissue depth of up to 6 mm (Fig. S7C). To determine the imaging resolution, the signal-to-background ratio (SBR) was calculated for each depth, and the full width at half maxima (FWHM) of the capillaries was measured. Even at a depth of 6 mm, an SBR of 3.27 was detected in the chicken breasts (Fig. S7D), indicating good image quality; the FWHM reached 0.99 mm, exhibiting high imaging resolution (Fig. S7E). These findings reveal that Pkr(IR-Ca/Pda-uPA)-cRGD exhibited exceptional performance, including good tissue penetration, SBR, and imaging resolution, all of which were crucial for its potential in vivo imaging.

### In vitro selective binding to AP

**Fig. 3.**
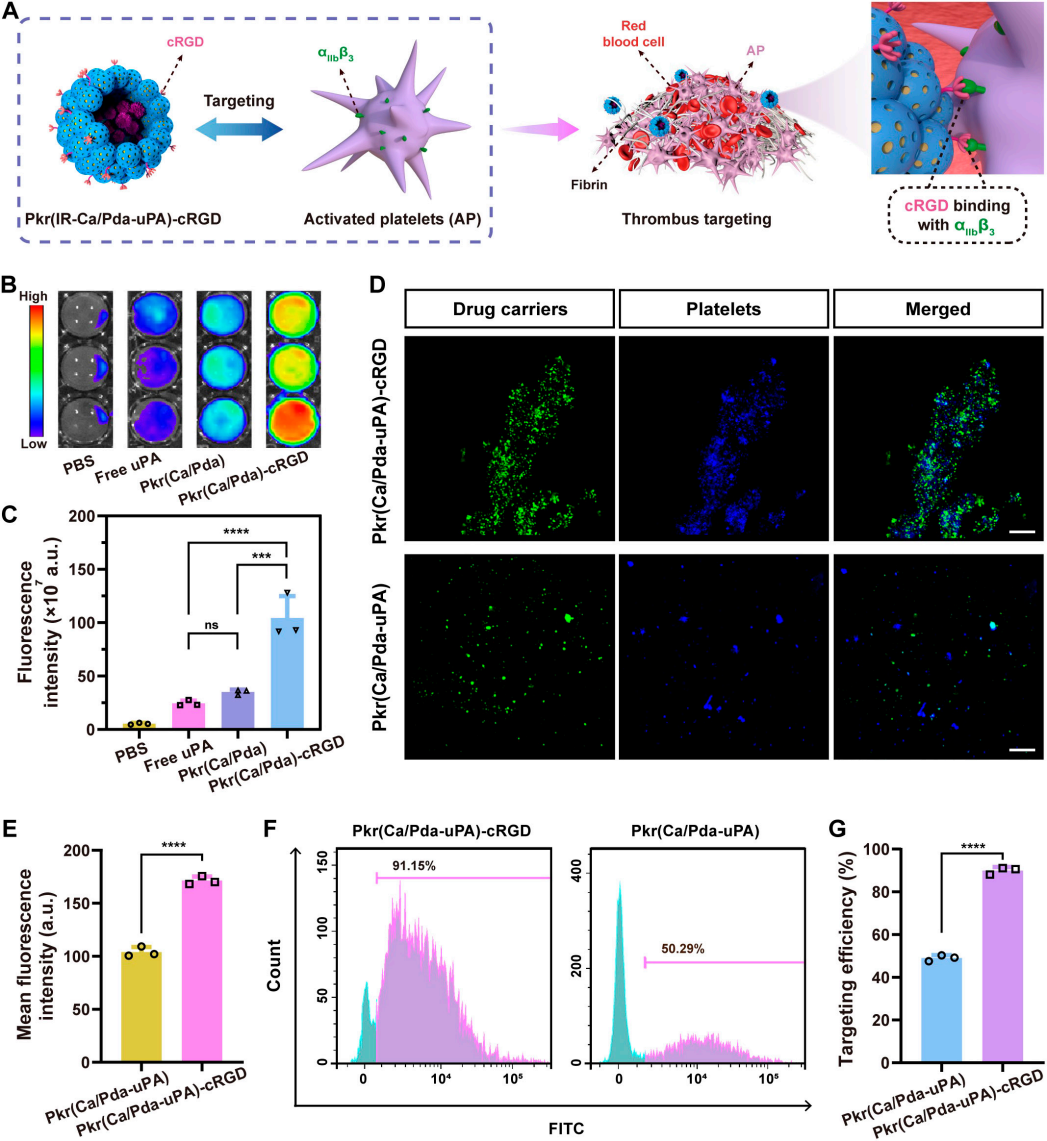
(A) Schematic of cRGD ligands targeted to α_IIb_β_3_ integrins from AP. (B) FLI of artificial blood clots incubated with various samples. (C) Fluorescence intensity of artificial blood clots. (D) CLSM of CD41a-eFluor 450-labeled AP incubated with FITC-labeled materials (scale bar: 10 μm). (E) Mean fluorescence intensity of FITC-labeled materials. (F) Binding rate to AP and (G) the corresponding fluorescence-activated cell sorting analysis. Statistical analysis for (C) was performed using the ANOVA (multiple comparisons) test, while the Student *t* test for (E) and (G). Data are presented as the mean ± SD (*n* = 3). ***P < 0.001, ****P < 0.0001, and ns represents no significant difference between 2 groups.

GPIIb-IIIa integrins are highly expressed on the surface of AP, making them attractive targets for selective binding by cRGD peptides. Therefore, Pkr(IR-Ca/Pda-uPA)-cRGD was decorated with cRGD for targeting to AP from thrombi for precise drug delivery to thrombi (Fig. 3A). Initially, the capability of static targeting was evaluated using artificial blood clots to simulate thrombi. The fluorescent signals in artificial blood clots were evaluated following coincubation with phosphate-buffered saline (PBS) (control), Pkr(Ca/Pda), Pkr(Ca/Pda)-cRGD, and free uPA. The fluorescence images of the clots and fluorescence intensity revealed that Pkr(Ca/Pda)-cRGD had the strongest fluorescence signals, indicating its effective accumulation at the thrombi (Fig. 3B and C). Therefore, it can be inferred that grafting cRGD had markedly improved the thrombus-targeting ability of Pkr(Ca/Pda) without uPA. Furthermore, to determine whether the cRGD peptide enhanced the selective binding ability of Pkr(Ca/Pda-uPA)-cRGD carrying uPA, confocal laser scanning microscopy (CLSM) with CD41a-eFluor 450 as the AP marker was used. It demonstrated that Pkr(Ca/Pda-uPA)-cRGD had higher binding ability than that of Pkr(Ca/Pda-uPA) (Fig. 3D), with about 1.7-fold higher selective binding to AP (Fig. 3E). Furthermore, colocalization analysis was performed to validate that the addition of cRGD peptide improved Pkr(Ca/Pda-uPA)-cRGD’s preferential binding to thrombi. Specific and close contacts were observed between the cRGD peptides (stained by Cy5.5) from Pkr(Ca/Pda-uPA)-cRGD and the αIIbβ3 integrins (stained by CD41a) from AP (Fig. S8). The overlap of the 2 fluorescence signals in the merged image provided strong evidence that the cRGD peptides indeed improved the binding specificity of Pkr(Ca/Pda-uPA)-cRGD.

Additionally, flow cytometric study revealed that Pkr(Ca/Pda-uPA)-cRGD was more selective (binding rate: 91.2%) than Pkr(Ca/Pda-uPA) (binding rate: 50.3%) (Fig. 3F and G), further confirming that grafting cRGD greatly enhanced the thrombus-targeting ability of Pkr(Ca/Pda-uPa)-cRGD carrying uPA, which was similar to that of Pkr(Ca/Pda)-cRGD without uPA (Fig. 3B and C).

**Fig. 4.**
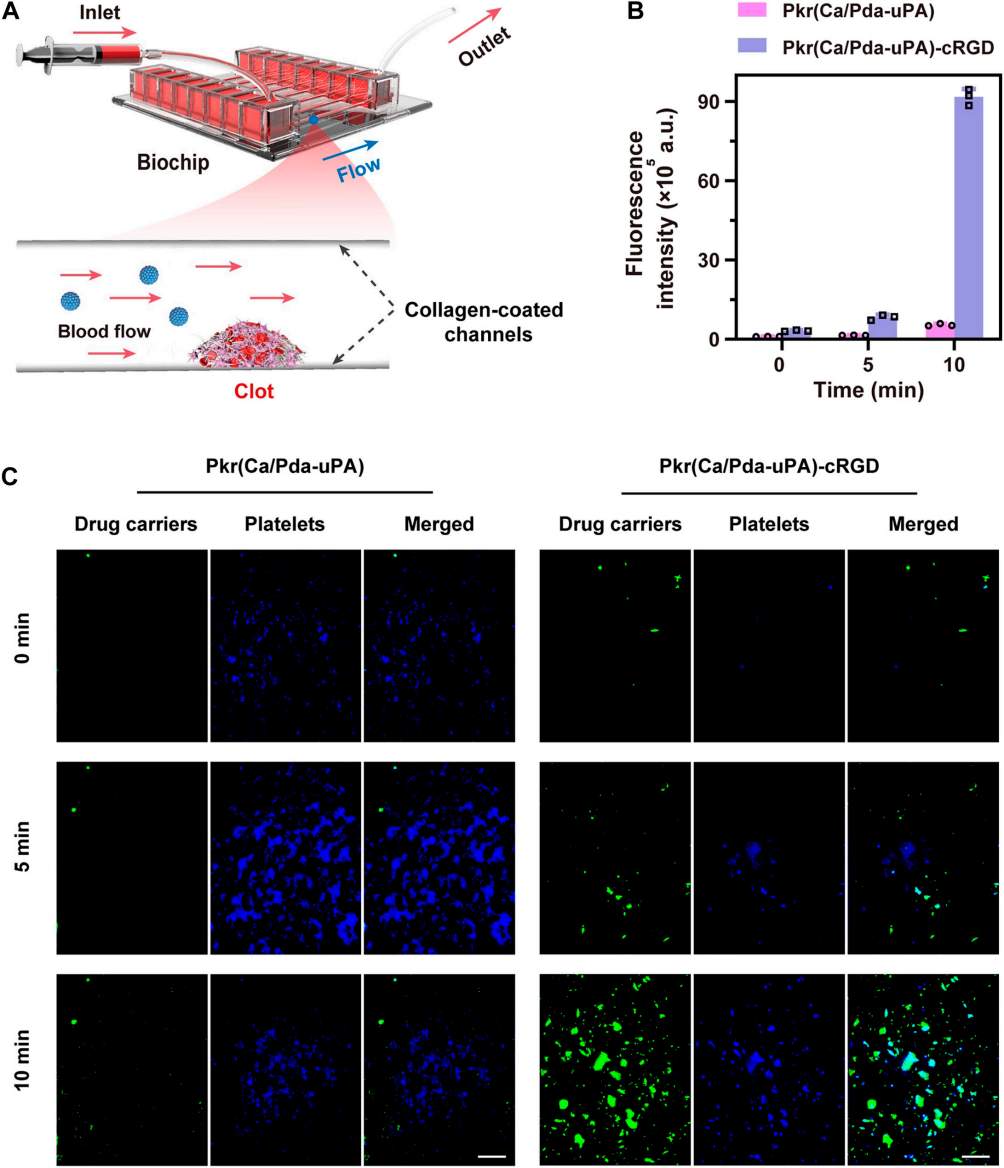
(A) Schematic of the microfluidic system. (B) Fluorescence intensity of FITC-labeled drug carriers bond to stimulated thrombi. (C) Fluorescence images of FITC-labeled drug carriers bond to stimulated thrombi (scale bar: 10 μm). All error bars depicted the mean ± SD of at least 3 independent experiments.

Next, the dynamic targeting ability of Pkr(Ca/Pda-uPA)-cRGD to thrombi was investigated using a microfluidic system (Fig. 4A). The device consists of interconnected channels that were designed to mimic the size and geometry of blood vessels. Consequently, the fluid flow closely resembles the physiological conditions of the circulatory system, allowing to assess the targeting efficiency and selectivity of Pkr(Ca/Pda-uPA)-cRGD more accurately. Within the microfluidic channels, fluorescein isothiocyanate (FITC)-labeled Pkr(Ca/Pda-uPA)-cRGD and Pkr(Ca/Pda-uPA) in solution were allowed to flow into the channels to meet the stimulated thrombus. Fluorescence intensity of FITC in stimulated thrombi was used to evaluate targeting capacity. It was found that Pkr(Ca/Pda-uPA)-cRGD possessed higher fluorescence density (Fig. 4C), which was decoupled to that of Pkr(Ca/Pda-uPA) (Fig. 4B), suggesting its superior selectivity to target thrombi.

### In vitro pH-responsive release of uPA

**Fig. 5.**
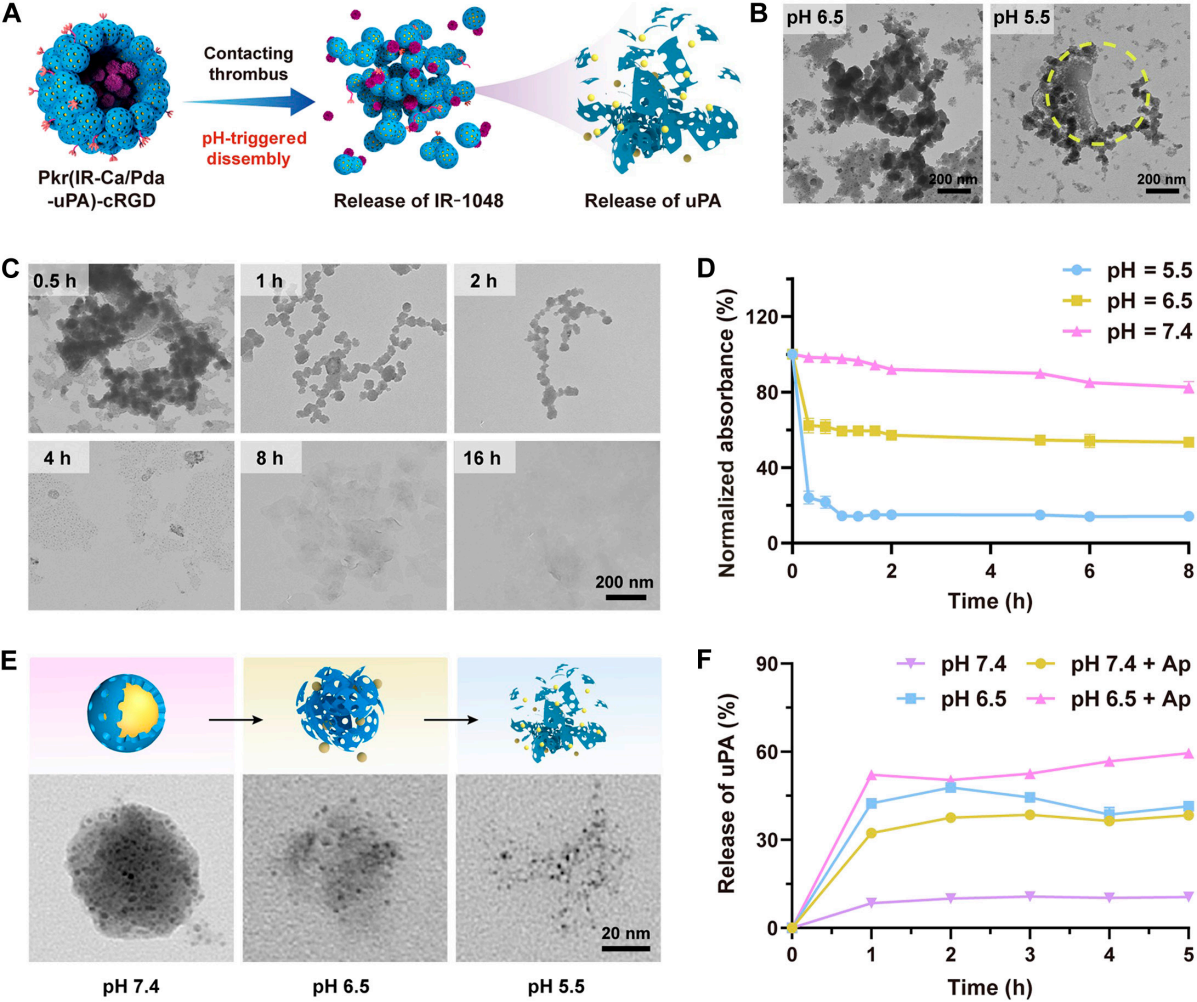
(A) pH-responsive disintegration of Pkr(IR-Ca/Pda-uPA)-cRGD and dissolution of CaCO_3_-PDA. (B) Disintegration of Pkr(IR-Ca/Pda-uPA)-cRGD at different pH. (C) Time-dependent disintegration of Pkr(IR-Ca/Pda-uPA)-cRGD. (D) Time-dependent absorbance of CaCO_3_-PDA. (E) Decomposition of CaCO_3_-PDA at different pH. (F) Release of uPA by Pkr(Ca/Pda-uPA)-cRGD after incubation with resting platelets or APs. All error bars depicted the mean ± SD of at least 3 independent experiments.

The pH-triggered disintegration of Pkr(IR-Ca/Pda-uPA)-cRGD is a crucial process to release IR-1048 dye and uPA for theranostic of thrombi after targeting. Figure 5A demonstrates the schematic disintegration of Pkr(IR-Ca/Pda-uPA)-cRGD at a low pH, which may be offered by the physiological environment around thrombi. To investigate the pH-responsive disintegration, Pkr(IR-Ca/Pda-uPA)-cRGD was immersed in PBS at pH 5.5 and pH 6.5 before transmission electron microscopy (TEM) observation. Pkr(IR-Ca/Pda-uPA)-cRGD particles became destabilized at pH 6.5 and considerably disintegrated at pH 5.5 (Fig. 5B). Figure 5C illustrates the time-dependent dynamic disintegration of Pkr(IR-Ca/Pda-uPA)-cRGD at pH 6.5. Pkr(IR-Ca/Pda-uPA)-cRGD struggled for the first 0.5 h to preserve its spherical shape but gradually disintegrated over time. Similar phenomenon could also be found in pH-responsive drug carriers developed by Zheng et al. [40] and Lu et al. [41]. The disintegration of Pkr(IR-Ca/Pda-cRGD) shall be linked to the instability of the PDA backbone at pH 6.5. Following the disintegration of Pkr(IR-Ca/Pda-uPA)-cRGD, CaCO_3_-PDA continued to dissolve as time passed or pH decreased further. CaCO_3_-PDA was shown to be stable at pH 7.4 but dissolved at pH 6.5 and pH 5.5. (Fig. 5E); its ultraviolet-visible (UV-vis) absorbance declined as pH decreased (Fig. 5D), further suggesting that CaCO_3_-containing materials were extremely pH-sensitive. The pH-responsive release rate of uPA by Pkr(Ca/Pda-uPA)-cRGD was much larger at pH 6.5 (42.0% in 1 h) than at pH 7.4 (8.0% in 1 h) (Fig. 5F). In addition, the presence of AP (pH 7.4 + AP and pH 6.5 + AP) resulted in an even greater release rate of uPA (52.1% at pH 6.5 in 1 h), indicating that the release of uPA was attributed to both Pkr(Ca/Pda-uPA)-cRGD’s pH responsiveness and selective binding capacity to AP. This could be supported by published studies [42,43].

### In vitro evaluation of fibrinolysis and thrombolysis

In thrombolysis, uPA can convert plasminogen into plasmin to trigger fibrinolysis for dissolution of fibrin in blood clots. To examine the dynamic fibrinolytic performance of Pkr(Ca/Pda-uPA)-cRGD in vitro, absorbance measurements were first used to track the content of fibrin. More fibrin is present in the incubation mixture as absorbance increases. After 60 min of incubation with thrombin, the absorbance increased steadily, indicating that fibrinogen had been transformed to fibrin (Fig. S9). Followed by addition with Pkr(Ca/Pda-uPA)-cRGD at 60 min, absorbance decreased in all groups except for the blank control, suggesting that the released uPA from Pkr(Ca/Pda-uPA)-cRGD induced fibrinolysis to dissolve fibrin in the incubation mixture. In addition, it was noted that absorbance decreased very slightly at pH 7.4, whereas absorbance dropped markedly at pH 6.5. This was because disintegration of Pkr(Ca/Pda-uPA)-cRGD occurred at pH 6.5 to release uPA for fibrinolysis, whereas structurally stable Pkr (Ca/Pda-uPA)-cRGD hardly released uPA at pH 7.4. Lastly, the fibrinolysis rate rose dramatically when AP was added (pH 7.4 + AP and pH 6.5 + AP group) due to the pH responsiveness of Pkr(Ca/Pda-uPA)-cRGD and its selective binding capacity to AP, both of which contributed to enhanced uPA release. This was in line with the uPA release behavior investigated earlier (Fig. 5E).

**Fig. 6.**
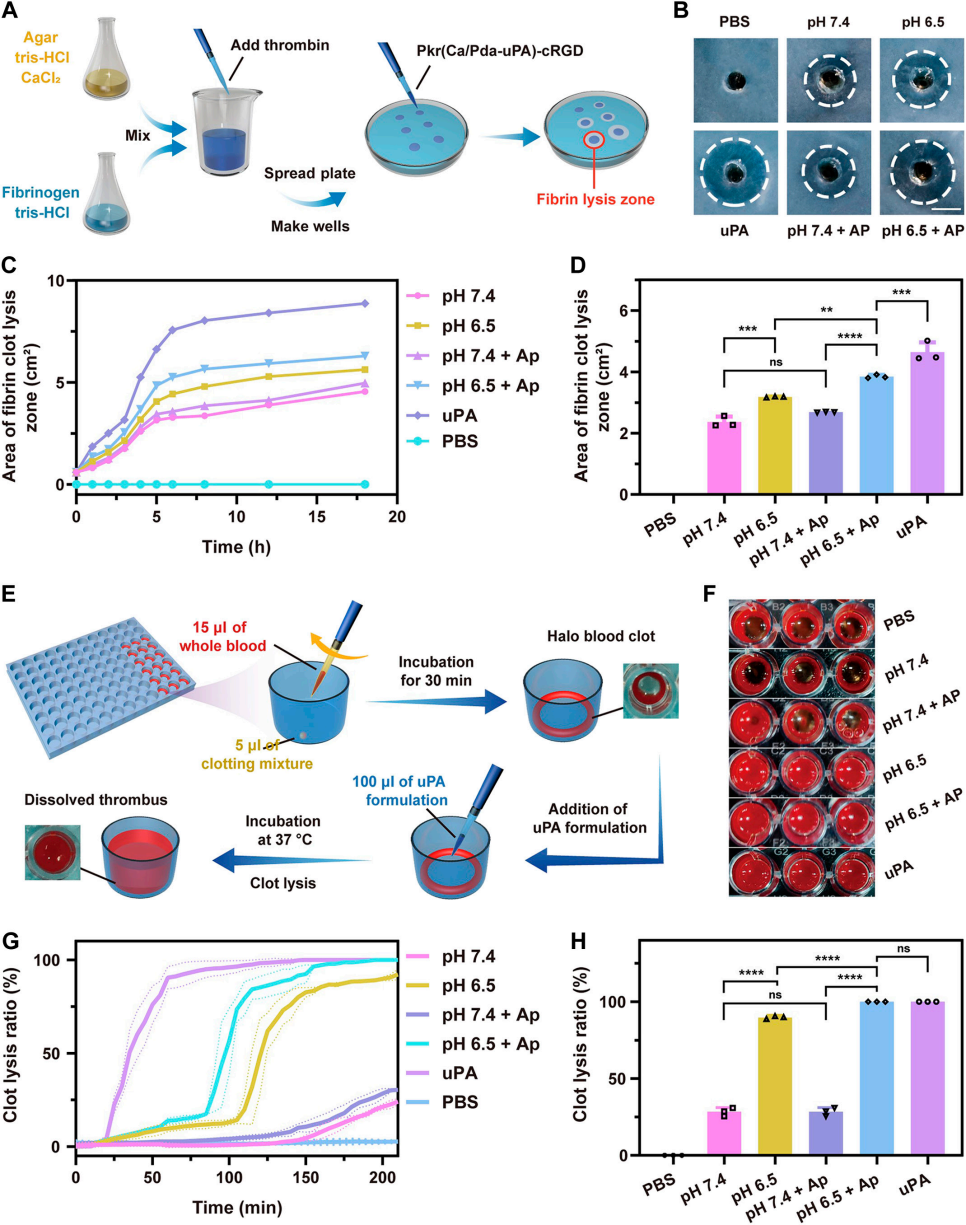
(A) Schematic of the agar plate model. (B) Representative photos of fibrin clots after 240 min of treatment (scale bar: 1 cm). (C) Time-dependent area of fibrin clot lysis zone. (D) Comparison of area of fibrin clot lysis zone after 240 min of treatment. (E) Schematic of the halo blood clot model. (F) Clots treated with various uPA formulation. (G) Time-dependent of clot lysis. (H) Comparison of clot lysis ratio after 200 min of treatment. Statistical analysis for (D) and (H) was performed using the ANOVA (multiple comparisons) test. Data are presented as the mean ± SD (*n* = 3). **P < 0.01, ***P < 0.001, ****P < 0.0001, and ns represents no significant difference between 2 groups.

The fibrinolytic activity of Pkr(Ca/Pda-uPA)-cRGD was then evaluated by the agar plate assay (Fig. 6A), in which the area of fibrin lysis zone was tested. Fibrin clots treated with Pkr(Ca/Pda-uPA)-cRGD displayed clear fibrin lysis zone after 4 h (Fig. 6B), whereas those treated with PBS alone did not exhibit fibrinolysis even after 18 h (Fig. 6C). The fibrinolytic activity of Pkr(Ca/Pda-uPA)-cRGD varied based on the treatment conditions. Specifically, fibrinolysis at pH 6.5 (area of the lysis zone: 3.18 cm^2^) was larger than that at pH 7.4 (area of the lysis zone: 2.36 cm^2^) (Fig. 6D), which was because the dissolution of CaCO_3_-PDA-uPA at pH 6.5 predominated over that at pH 7.4. The lower pH promoted CaCO_3_-PDA-uPA to release uPA for fibrinolysis. Similarly, fibrinolysis in the presence of AP was shown to be further accelerated, corroborating the findings in Fig. 5F and Fig. S8 that the interaction of AP with cRGD stimulated the release of uPA from Pkr(Ca/Pda-uPA)-cRGD. This is consistent with previously reported studies [39].

The thrombolytic activity of Pkr(Ca/Pda-uPA)-cRGD was further evaluated in a halo blood clot model (Fig. 6E). Pkr(Ca/Pda-uPA)-cRGD was added into a well containing a halo-shaped blood clots, which was then incubated at 37 °C for clot lysis by uPA released from Pkr(Ca/Pda-uPA)-cRGD. The absorbance of the mixture in the well was then measured to evaluate the thrombolytic effectiveness. Increased absorbance indicated enhanced clot lysis. The PBS and free uPA groups were employed as controls. Halo clots were entirely dissolved by free uPA (Fig. 6F); however, only 28.35% of halo clots dissolved by Pkr(Ca/Pda-uPA)-cRGD at pH 7.4 for 200 min (Fig. 6G). Notably, 89.82% dissolution was recorded at pH 6.5, and the presence of AP enhanced dissolution further (Fig. 6H). These findings suggested that Pkr(Ca/Pda-uPA)-cRGD displayed pH-responsive thrombolytic activity, which was in a good agreement with fibrinolysis.

### In vitro dynamic targeted thrombolysis

Prior research has evaluated the thrombolytic activity of Pkr(Ca/Pda-uPA)-cRGD under static settings. A microfluidic system was used to assess its dynamic efficacy under physiological flow parameters. 3,3’-Dihexyloxacarbocyanine iodide (DIOC6)-labeled fresh mouse blood (stained on platelets) was perfused through the collagen-coated channels (Fig. 4A) to induce nonocclusive DIOC6-labeled thrombus. The thrombi were then treated with Pkr(Ca/Pda)-cRGD, Pkr(Ca/Pda-uPA)-cRGD, or free uPA before observation (Fig. S10A). Platelet fluorescence nearly disappeared after 16 min of perfusion with Pkr(Ca/Pda-uPA)-cRGD; however, there were no changes in fluorescence when Pkr(Ca/Pda-cRGD) was utilized. This demonstrated that critical clot lysis occurred in the presence of released uPA from Pkr(Ca/Pda-uPA)-cRGD, similar to the group treated with free uPA perfusion. Further, the time required to eliminate clot completely by Pkr(Ca/Pda-uPA)-cRGD was shorter than that of free uPA (Fig. S10B). This could be attributed to the targeting of Pkr(Ca/Pda-uPA)-cRGD to clot, which facilitated precise delivery and effective accumulation of uPA to the clot for promoted thrombolysis. The improved uPA delivery by Pkr(Ca/Pda-uPA)-cRGD also explained why clot loss was greater for Pkr(Ca/Pda-uPA)-cRGD (97.6%) than for free uPA (89.8%) (Fig. S10C).

### Biosafety evaluation

**Fig. 7.**
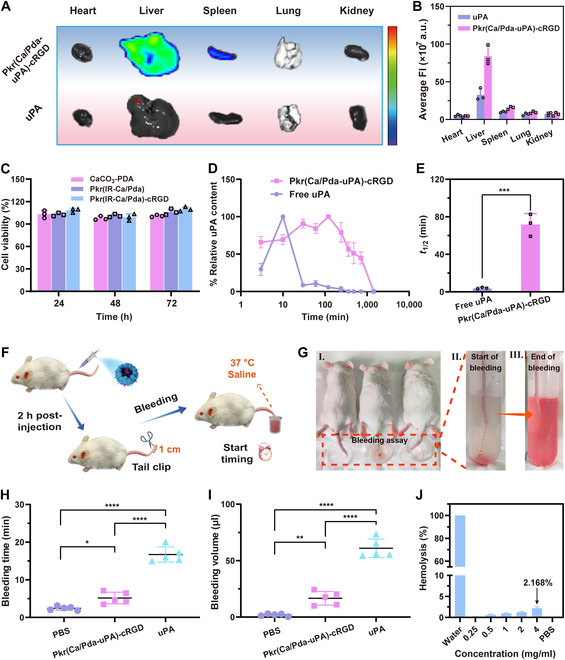
(A) FLI of mouse organs after intravenous injection (1.5 h later). (B) Fluorescence intensity of organs. (C) Cell viability of HUVECs. (D) Pharmacokinetic profiles of uPA. (E) Half-life times (*t*_1/2_) of uPA. (F) Schematic of tail bleeding risk assay. (G) I. Hemorrhage assay performed by truncating the tail tip, followed by immediate immersion in 37 °C saline solution. II. Beginning of bleeding. III. End of bleeding. (H) Bleeding time. (I) Bleeding volume. (J) Hemolytic activity of Pkr(IR-Ca/Pda)-cRGD. Statistical analysis for (H) and (I) was performed using the ANOVA (multiple comparisons) test, while the Student *t* test for (E). Data are presented as the mean ± SD (*n* = 3). *P < 0.05, **P < 0.01, ***P < 0.001, and ****P < 0.0001.

To examine the biodistribution of Pkr(Ca/Pda-uPA)-cRGD and uPA in the major organs of mice, imaging of Cy5.5-labeled uPA in various organs was performed following dissection. After 1.5 h, Pkr(Ca/Pda-uPA)-cRGD was observed to be primarily distributed in the liver, as indicated in Fig. 7A and B. The biodistribution in other organs was minimal and did not differ significantly. In contrast, free uPA was undetectable in all organs, indicating that uPA delivered without drug carriers was more likely to be eliminated from the body than uPA delivered with drug carriers. In a previous study, a similar finding was also observed [44]. Pkr(Ca/Pda-uPA)-cRGD was gradually eliminated over time, with only a small amount of fluorescent signal present in the liver after 24 h (Fig. S11A and B), suggesting that the drug carrier can be metabolized and excreted to avoid potential long-term toxicity. This suggested that uPA loaded in Pkr(Ca/Pda-uPA)-cRGD was superior to free uPA for drug delivery toward precise targeting, since the therapeutic efficacy of uPA was substantially conserved.

The biosafety was further evaluated using Cell Counting Kit-8 cytotoxicity assays and live/dead cell staining procedures. The materials had minimal effect on the viability of human umbilical vein endothelial cells (HUVECs) in comparison to the control group (Fig. 7C), which can be supported by the live/dead staining of HUVECs (Fig. S12). In addition, the hemolysis rate was all less than 5%, regardless of Pkr(IR-Ca/Pda)-cRGD concentration (Fig. 7J). In addition, immunoglobulin level evaluation was not significantly different compared to the control group (Fig. S13A), indicating that Pkr(IR-Ca/Pda-uPA)-cRGD did not induce strong inflammation reaction and immune rejection response after intravenous administration; in addition, the evaluation of blood biochemical indexes, blood routine indexes, and immune response (immunoglobulin G [IgG] and IgM) further demonstrated that there was no significant difference in blood parameters after administration of Pkr(IR-Ca/Pda-uPA)-cRGD (Fig. S13B). The results led to the conclusion that Pkr(IR-Ca/Pda)-cRGD had good biocompatibility and was safe as a drug carrier.

### Pharmacokinetics

The insufficient duration of drug circulation in the bloodstream can have a negative effect on the efficacy of thrombolysis. In order to increase thrombus-specific accumulation and lengthen circulation time, uPA was encapsulated in the drug carrier for this investigation. To determine the circulation duration, the fluorescence intensity of FITC-uPA was used to evaluate the uPA concentration in the cardiac blood of rats injected intravenously with either FITC-labeled free uPA or FITC-labeled Pkr(Ca/Pda-uPA)-cRGD (corresponding to a dose of 10 mg kg^−1^). Pkr(Ca/Pda-uPA)-cRGD had a longer retention period than free uPA (Fig. 7D). The circulating half-life of Pkr(Ca/Pda-uPA)-cRGD was up to 72 min, whereas that of free uPA was as low as 4 min (Fig. 7E), showing that free uPA was rapidly removed from the circulation after intravenous injection. Due to the prolonged circulation duration, Pkr(Ca/Pda-uPA-cRGD was believed to effectively target and penetrate thrombi to promote thrombolytic therapy.

### Tail bleeding risk assessment

Systemic injection of free uPA may increase the danger of unintended bleeding, whereas uPA encapsulated in drug carriers was expected to eliminate this risk. The tail bleeding test was used to evaluate the efficacy of Pkr(Ca/Pda-uPA)-cRGD in preventing unintended bleeding during uPA-induced thrombolytic treatment. The danger of bleeding increases with the duration of tail bleeding and the bleeding volume. After the injection of PBS, Pkr(Ca/Pda-uPA)-cRGD, or free uPA into mice tail, the distal tip of the tail was snipped (1 cm in length) to induce bleeding (Fig. 7F and G). As demonstrated in Fig. 7H, mice treated with Pkr(Ca/Pda-uPA)-cRGD achieved hemostasis in approximately 5 min, which was only one-third of that to the group treated with free uPA (approximately 16 min). Moreover, the bleeding volume induced by free uPA was substantially greater than that induced by Pkr(Ca/Pda-uPA)-cRGD (Fig. 7I). These results suggested that encapsulating uPA in Pkr(Ca/Pda-uPA)-cRGD successfully reduced the risk of unintentional bleeding associated with free uPA during thrombolytic therapy.

### In vivo thrombolysis in mouse tail venous thrombosis model

**Fig. 8.**
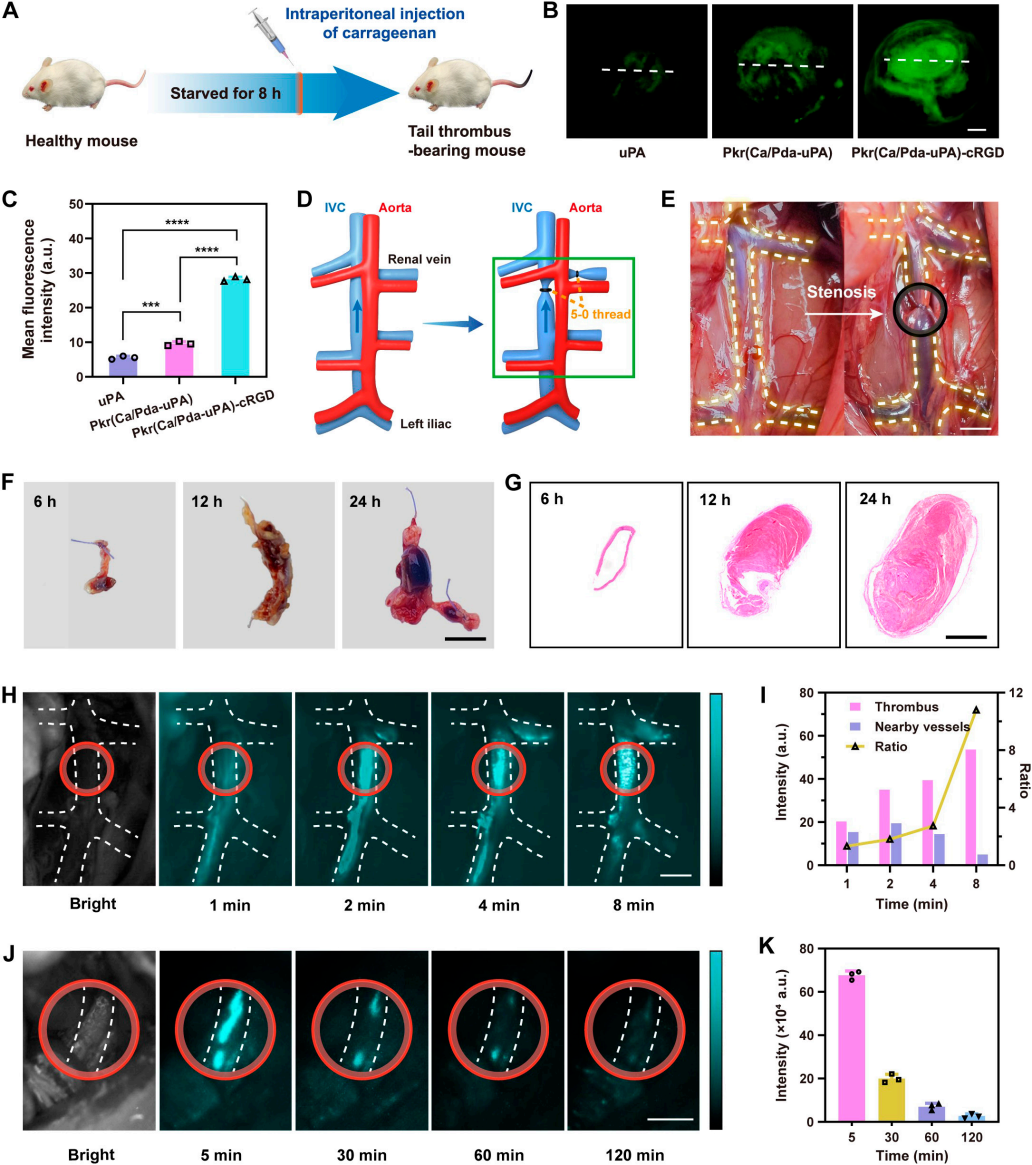
(A) Establishment of the tail thrombus model. (B) Fluorescent images of tail thrombi (scale bar: 100 μm). (C) Mean fluorescence intensity of tail thrombi. (D) Schematic to establish DVT model by IVC stenosis. (E) IVC stenosis-induced DVT model (the black circle spotlighted the stenosis site, scale bar: 5 mm). (F) Isolated thrombotic IVC (scale bar: 5 mm). (G) Hematoxylin and eosin-stained histological sections of thrombotic IVC (scale bar: 1 mm). (H) In vivo real-time NIR-II FLI of thrombi of DVT with Pkr(IR-Ca/Pda)-cRGD. The white dashed lines depict the corresponding part of thrombotic IVC, and red circles spotlight thrombi in thrombotic IVC (scale bar: 5 mm). (I) Fluorescence intensity of thrombi in thrombotic IVC (treated with Pkr(IR-Ca/Pda)-cRGD) and the nearby vessels. (J) In vivo real-time NIR-II FLI of thrombi of DVT during thrombolysis with Pkr(IR-Ca/Pda-uPA)-cRGD. The white dashed lines depict thrombotic IVC, and red circles spotlight thrombi in corresponding part of thrombotic IVC (scale bar: 5 mm). (K) NIR-II fluorescence intensity of thrombi in thrombotic IVC. Statistical analysis for (C) was performed using the ANOVA (multiple comparisons) test. Data are presented as the mean ± SD (*n* = 3). ***P < 0.001 and ****P < 0.0001.

After confirming that Pkr(Ca/Pda-uPA)-cRGD reduced the risk of unintentional bleeding in uPA-induced thrombolytic therapy, the in vivo thrombolytic capacity of Pkr(Ca/Pda-uPA)-cRGD was firstly evaluated in a mouse tail venous thrombus model. As shown in Fig. 8A, the model was established by injecting fresh carrageenan (20 mg kg^−1^) intraperitoneally into Kunming (KM) mice that had been fasting for 8 h. Mice with similar length of tail thrombi (approximately 4.5 cm) was then used for treatment with PBS, Pkr(Ca/Pda-uPA), Pkr(Ca/Pda-uPA)-cRGD, and free uPA (Fig. S14). The length of the tail thrombus in mice that received treatment (Fig. S15A) was compared to that of mice prior to treatment. The control group treated with PBS and free uPA showed an increase in thrombus length, to 5.7 and 4.8 cm, respectively (Figs. S15B and S16A). In contrast, the groups treated with Pkr(Ca/Pda-uPA) and Pkr(Ca/Pda-uPA)-cRGD inhibited thrombus growth due to the thrombolytic effect of the carried uPA, demonstrating the importance of drug carriers for uPA delivery. Specifically, the Pkr(Ca/Pda-uPA)-cRGD group demonstrated a greater decrease in tail thrombus length (1.61 cm) than the Pkr(Ca/Pda-uPA) group (1.15 cm) (Fig. S16B), which could be ascribed to the efficient targeting capacity of Pkr(Ca/Pda-uPA)-cRGD contributed to the precise release of uPA at thrombus sites.

In addition to measuring the length of tail thrombi, histological analysis was performed to validate the thrombolytic efficacy of Pkr(Ca/Pda-uPA-cRGD. Thrombolysis hardly occurred in mice treated with PBS and free uPA, as thrombi almost entirely filled the blood vessel (Fig. S17). This may be a result of the quick clearance of free uPA from circulation and the nonthrombus targeting of uPA. In contrast, treatment with Pkr(Ca/Pda-uPA) and Pkr(Ca/Pda-cRGD) remarkably reduced thrombus size. Notably, Pkr(Ca/Pda-uPA)-cRGD demonstrated better thrombolytic effectiveness in comparison to Pkr(Ca/Pda-uPA), with full thrombus breakdown and vascular unblocking, owing to its increased thrombus targeting. This finding further verified the thrombolytic potential of Pkr(Ca/Pda-uPA)-cRGD as a targeted thrombolytic agent.

Although Pkr(Ca/Pda-uPA-cRGD had preliminarily displayed potentials in thrombolysis, the interactions between Pkr(Ca/Pda-uPA-cRGD microparticles and thrombi substrate were still uncertain. Due to the low diffusion of thrombolytic medicines into thrombus substrate, the efficiency of thrombolysis may be questioned, especially for deep vein thrombi. In particular, the dense fibrin meshwork within the thrombus may impede drug penetration, decreasing drug delivery efficiency and posing risks in incomplete thrombolysis or re-embolism. To tackle this, a previous study utilized magnetically driving and ultrasound stimulation to enhance thrombus-penetrating capacity of drugs [16].

In order to comprehend the ability of Pkr(Ca/Pda-uPA)-cRGD microparticles to penetrate thrombus substrates, FLI and histochemical investigations of a mouse tail thrombus model were conducted. Fluorescence microscopy was used to view histological sections of mouse tail thrombi treated with FITC-labeled free uPA, Pkr(Ca/Pda-uPA), and Pkr(Ca/Pda-uPA)-cRGD. As shown in Fig. 8B and C, free uPA-treated thrombi showed ultraweaker fluorescence in general, which distributed mainly at the thrombus edge only, but thrombi treated with FITC-labeled Pkr(Ca/Pda-uPA)-cRGD and Pkr(Ca/Pda-uPA) exhibited much stronger fluorescence. The ultraweak fluorescence of the free uPA group may be attributable to (a) the low delivery rate of uPA to the thrombus site due to the short half-life of free uPA in the blood, (b) the low selective binding of free uPA to the thrombus, and/or (c) the poor penetration of free uPA into the thrombus substrate. Additionally, fluorescence spread from the outside to the inside of thrombi in both the Pkr(Ca/Pda-uPA)-cRGD and Pkr(Ca/Pda-uPA) groups, but the proportion of fluorescence distribution varied. Specifically, the fluorescence intensity in the Pkr(Ca/Pda-uPA) group was negligible in the interior portion of the thrombus substrate, whereas the fluorescence intensity in the Pkr(Ca/Pda)-cRGD group was visible in the inner portion of the thrombus substrate (Fig. S18). This could be explained that cRGD ligands decorated over Pkr(Ca/Pda-uPA)-cRGD surface improved targeted delivery of uPA to thrombus sites by Pkr(Ca/Pda-uPA)-cRGD and promoted penetration of Pkr(Ca/Pda-uPA)-cRGD into thrombus substrates to release uPA. This merit confirmed the potentials of Pkr(Ca/Pda-uPA)-cRGD in DVT therapy.

### In vivo assessment of targeted thrombolysis in DVT model

In addition to the preliminary evaluation of thrombolysis in the mouse tail venous thrombosis model, the in vivo thrombolytic capacity of Pkr(IR-Ca/Pda-uPA)-cRGD was assessed utilizing the rat inferior vena cava (IVC) stenosis-induced DVT model to illustrate the clinical translation potential, as IVC stenosis permits residual blood flow and does not cause endothelial injury. As shown in Fig. 8D and E, the IVC was stenosed with 5-0 sutures near the left renal vein at 5 mm, and all visible side branches between the left renal vein and the iliac vein were ligated to construct the thrombotic IVC for the DVT model. Following stenosis for varied periods, representative digital photos (Fig. 8F) and histological sections (Fig. 8G) of the IVC revealed the evolution of thrombus in IVC stenosis-induced DVT. The DVT model created by 12-h stenosis was used for subsequent NIR-II FLI of thrombi in vivo to monitor the targeted thrombolytic process in real time.

Figure 8H displays the real-time NIR-II FLI of DVT thrombi in vivo following caudal vein injection of Pkr(IR-Ca/Pda)-cRGD (without uPA). It was obvious that the fluorescence signal in the IVC increased dramatically over time, suggesting that Pkr(IR-Ca/Pda)-cRGD progressively anchored into thrombi of DVT and accumulated massively over time due to its exceptional targeting ability. Whereas the fluorescence signal around the thrombus persisted at a relative lower level, independent of time, demonstrating that no preferential binding of Pkr(IR-Ca/Pda)-cRGD onto the vessels occurred. After 8 min of Pkr(IR-Ca/Pda)-cRGD injection, the peak ratio of the fluorescence signal intensity difference between the thrombus and adjacent blood vessels was 10.8 (Fig. 8I). This shows that Pkr(IR-Ca/Pda)-cRGD could be employed for real-time NIR-II FLI of in-progress thrombus formation in DVT diagnosis prior to therapy, which is crucial for the targeted treatment of DVT.

Similarly, Pkr(IR-Ca/Pda-uPA)-cRGD, which was prepared from encapsulation of uPA by Pkr(IR-Ca/Pda)-cRGD, should also be capable for real-time NIR-II FLI of in-progress thrombus elimination in thrombolytic therapy of DVT. To confirm this, the dynamic in vivo thrombolytic process of DVT using Pkr(IR-Ca/Pda-uPA)-cRGD was tracked further by NIR-II FLI. As displayed in Fig. 8J, NIR-II stimuli after 5 min of Pkr(IR-Ca/Pda-uPA)-cRGD injection clearly imagined the thrombus of DVT presenting in the IVC. After that, a progressive drop in thrombus fluorescence intensity was seen over time, indicating that the thrombus was gradually cleared. Consequently, it was apparent that NIR-II FLI could accurately observe the progression of thrombolysis in real time, which is typically unobservable with general optical imaging (Fig. S19A). The thrombolytic process could be summarized as follows: initially, a distinct fluorescence signal developed at the thrombus after intravenous injection of Pkr(IR-Ca/Pda-uPA)-cRGD, demonstrating targeted aggregation of Pkr(IR-Ca/Pda-uPA)-cRGD into the thrombus; then, Pkr(IR-Ca/Pda-uPA)-cRGD began to disintegrate due to the low pH features of the thrombus, releasing IR-1048 dye for imaging; eventually, individual CaCO_3_-PDA began to self-dissolve concurrently with the disintegration of Pkr(IR-Ca/Pda-uPA)-cRGD, releasing the thrombolytic medication uPA for successful thrombolysis. After 120 min of therapy, there was little fluorescence at the thrombus, indicating that the vast bulk of the thrombus had been eliminated. This was also validated by a histological analysis of the thrombus and vessel cross-sections (Fig. S19B). Therefore, the NIR-II FLI of thrombi represented the actual status of thrombi in vessels during DVT therapy, which is anticipated to provide accurate real-time information on thrombi in order to minimize undertreatment and overtreatment risks of thrombosis.

## Conclusion

Thrombi are unintentionally formed blood clots that can obstruct blood vessels and result in the majority of life-threatening disorders. Due to the off-target nature of thrombolytic medications, the current therapeutic treatment for thrombosis based on systemic injection of thrombolytic agents has been associated with unexpected bleeding complications. In addition, the present clinical diagnostic procedures for thrombosis are hindered by technological obstacles, resulting in a lack of diagnostic precision and convenience. Importantly, the lack of real-time visualization of thrombosis has raised concerns of overtreatment and undertreatment. This study developed a theranostic drug carrier, Pkr(IR-Ca/Pda-uPA)-cRGD, for real-time NIR-II visualization of targeted therapy of deep venous thrombosis. Material characterization confirmed that Pkr(IR-Ca/Pda-uPA)-cRGD, the colloidosome microparticle, carried both the hydrophilic thrombolytic drug (uPA) and hydrophobic contrast agent of NIR-II FLI (IR-1048 dye), which were exceptionally hard to encapsulate in one single-drug carrier. Flow cytometry and laser confocal microscopy proved the targeting capacity of Pkr(IR-Ca/Pda-uPA)-cRGD to thrombus, which could be ascribed to the inherent exclusive binding between cRGD from Pkr(IR-Ca/Pda-uPA)-cRGD and GPIIb-IIIa integrins from thrombus. Following targeting to thrombi, the acidic microenvironment triggered disintegration of Pkr(IR-Ca/Pda-uPA)-cRGD to release IR-1048 and uPA into thrombus substrate controllably and precisely for visualization of the targeted thrombolytic procedure. In the mouse caudal thrombosis model, Pkr(IR-Ca/Pda-uPA)-cRGD exhibited efficient targeting and penetration capacity to thrombus substrates to considerably eradicate tail venous thrombi (approximately 1.61 cm) and decreased the unintentional bleeding complications; additionally, the rat DVT model further confirmed that targeted thrombolysis was successfully visualized by NIR-II FLI in minutes, enabling real-time monitoring of thrombus ablation to avoid overtreatment and undertreatment risks. This theranostic drug carrier is a reliable integrated strategy for visualization of the targeted thrombolytic process with NIR-II, hence increasing clinical translation potential in real-time diagnosis during co-occurrence therapy of thrombosis.

## Materials and Methods

### Materials

BSA, fibrinogen, Serine Proteases Chromogenic Activity Assay Kit S-2288, DiOC6, NHS, 1-(3-dimethylaminopropyl)-3-ethylcarbodiimide (EDC·HCl), and IR-1048 dye were purchased from Sigma-Aldrich (Shanghai, China). CD41a-eFluor 450 were obtained from Thermo Fisher Scientific (Shanghai, China). cRGD peptides was purchased from Bioss Antibodies Reagent Co., Ltd. (Beijing, China). Urokinase-type plasminogen activator (uPA), agarose, thrombin, and adenosine diphosphate were obtained from Aladdin Reagent Co., Ltd. (Shanghai, China). The ultrafiltration centrifuge tube was purchased from Millipore (USA). Other reagents were all commercially available. All animal experiments were approved by the Animal Ethics Committee and performed following the protocols approved by the National Teaching Center of Animal Science and Experiment, Southwest University, China (accreditation number of the investigator: IACUC-20230918-07).

### Synthesis of CaCO_3_-PDA-uPA

CaCO_3_-PDA was prepared using a modified 1-pot gas diffusion method [38]. In brief, 200 mg of CaCl_2_ and 4 mg of dopamine were dissolved in 100 ml of anhydrous ethanol. The mixture was then placed in a sealed container with 5 g of NH_4_HCO_3_ and maintained at 37 °C. The bluish CaCO_3_-PDA was generated in the mixture after 24 h. Finally, CaCO_3_-PDA was dispersed in ethanol and stored at room temperature for later use. To encapsulate uPA for preparation of CaCO_3_-PDA-uPA, CaCO_3_-PDA (10 mg) was incubated with uPA solution (8,000 U ml^−1^, 1 ml) for 3 h. Then, CaCO_3_-PDA-uPA was collected and freeze-dried at −50 °C for 48 h. The encapsulation efficiency (EE%) and leakage rate of uPA in CaCO_3_-PDA-uPA were determined using the color-emitting substrate S-2288 technique [45]. The equation is as follows:

$$\mathrm{EE}\%=\left(1-\frac{\text{Amount of unencapsulated}\;uPA}{\text{Total amount of}\;uPA\;\text{initially used}}\right)\times 100$$

### Synthesis of Pkr(IR-Ca/Pda-uPA)

CaCO_3_-PDA-uPA (10 mg ml^−1^, 660 μl), IR-1048 dye (1 mg ml^−1^, 102 μl), and BSA solution (10 mg ml^−1^, 360 μl) were mixed under stirring at 7,500 rpm for 2 min, followed by standing overnight to produce Pkr(IR-Ca/Pda-uPA). For comparison, similar synthetic experiments may be carried out, in the absence of IR-1048, to prepare (a) Pkr(Ca/Pda) using CaCO_3_-PDA and (b) Pkr(Ca/Pda-uPA) using CaCO_3_-PDA-uPA. The standard working curve and the drug loading capacity (DL%) of IR-1048 was calculated following a reported method [46]. The equation is as follows:

$$\mathrm{DL}\%=\left(\frac{\text{Weight of}\;IR1048\;dye\;\text{encapsulated}}{\text{Total weight of the dried colloidosome microsphere}}\right)\times 100$$

### Synthesis of Pkr(IR-Ca/Pda-uPA)-cRGD

Decoration of cRGD over Pkr(IR-Ca/Pda-uPA) relied on an amidation reaction as described elsewhere [39]. NHS (5.0 mg) and EDC (10.0 mg) were dissolved in an aqueous solution (10 ml), which was then added with cRGD peptide (10.0 mg) to prepare the activated aqueous solution. Pkr(IR-Ca/Pda-uPA)-cRGD was then prepared by adding Pkr(IR-Ca/Pda-uPA) to the activated solution and agitating it for 8 h at 4 °C. The resulting solution was then rapidly filtered with ultrafiltration tubes (Amicon Ultra Centrifugal Filters, molecular weight cutoff: 3,000 Da) to remove possible by-products. Eventually, Pkr(IR-Ca/Pda-uPA)-cRGD powder was collected by lyophilization.

### Characterization

The physiochemical properties were evaluated using TEM (HT7800 from HITACHI), scanning electron microscopy (SU8020), Fourier transform infrared spectroscopy (Nicolet IS 10), and nuclear magnetic resonance spectroscopy (^1^H NMR, Bruker AV400NMR). Zeta potential was measured using a zeta potential analyzer (Malvern ZEV3600). Fluorescence spectra were obtained with a photoluminescence spectrometer, whereas UV-vis spectra were collected

### In vitro NIR-II FLI

Different concentration of Pkr(IR-Ca/Pda-uPA)-cRGD solution was first filled in test tubes to determine the optimum concentration for FLI using the NIR-II imaging system (Hengguang Zhiying Medical Technology Co., Ltd., China). Then, the effect of tissue thickness on imaging was then determined by placing Pkr(IR-Ca/Pda-uPA)-cRGD solutions (0.1 mg ml^−1^) in capillaries (1 mm in diameter) beneath chicken breasts of varying thicknesses. The SBR and FWHM were calculated according to a previous study [47,48].

### In vitro FLI for evaluation of thrombus-targeting

Platelet-rich plasma (150 μl) from Sprague-Dawley rats was treated with thrombin (0.1 U l^−1^) and CaCl_2_ (0.3 M) for 90 min on a 96-well plate to create blood clots that mimic thrombi. Clots were then incubated for 10 min with PBS, Cy5.5-labeled Pkr(IR-Ca/Pda-uPA), Cy5.5-labeled Pkr(IR-Ca/Pda)-cRGD, and free uPA. Finally, the fluorescence signal of clots was evaluated using an imaging equipment (IVIS Lumina Series III) after the clots were rinsed 3 times with fresh PBS (pH 7.4).

### In vitro flow cytometry for evaluation of selective binding to AP

FITC-labeled Pkr(Ca/Pda-uPA) or Pkr(Ca/Pda-uPA)-cRGD solution (approximately 1 million, 1 ml), platelet obtained from KM mice (approximately 50 million, 0.5 ml, CD41a-eFluor 450 staining), and CaCl_2_ (5 mM) were mixed to incubate at 37 °C for 20 min. Then, the aggregation of FITC-labeled Pkr(Ca/Pda-uPA) or Pkr(Ca/Pda-uPA)-cRGD on AP was assessed using a flow cytometer (Thermo Fisher Scientific, USA).

### CLSM for evaluation of selective binding to AP under stationary conditions

CD41a-eFluor 450-stained platelet (approximately 10 million, 2 ml) in test tubes were activated by thrombin (1 U ml^−1^, 100 μl) for 20 min and then incubated with FITC-labeled Pkr(Ca/Pda-uPA) or Pkr(Ca/Pda-uPA)-cRGD for 30 min in a shaker at 37 °C. The selective binding of FITC-labeled Pkr(Ca/Pda-uPA) or Pkr(Ca/Pda-uPA)-cRGD was then observed using CLSM (FV3000RS, OLYMPUS, Japan) on platelets that had been carefully rinsed twice with PBS. In addition, CD41a-eFluor 450-stained platelets were also incubated with Cy5.5-labeled Pkr(Ca/Pda-uPA)-cRGD (labeling Cy5.5 onto cRGD) for 30 min at 37 °C for subsequent colocalization analysis using CLSM.

### Microfluidic system for evaluation of selective binding to AP under dynamic conditions

A mixture of ADP (50 μg ml^−1^) and collagen-I solution (0.5 mg ml^−1^) coated Vena8 Fluoro+ BioChip (2.8 cm × 0.4 mm × 0.1 mm; Cellix Ltd., Ireland) overnight at 4 °C. Then, blood from mice was stained with CD41a-eFluor 450 and perfused in collagen-coated channels at a wall shear rate of 1,000 s^−1^ at 25 °C to generate nonocclusive thrombi. Eventually, FITC-labeled Pkr(Ca/Pda-uPA) or Pkr(Ca/Pda-uPA)-cRGD was perfused into the channels for 10 min, and the contained thrombus inside the channel was viewed with a fluorescence microscope (Eclipse Ni-E, Japan) to evaluate selective binding to thrombi.

### In vitro uPA release assay

Pkr(Ca/Pda-uPA)-cRGD was introduced initially to PBS at pH 7.4 and pH 6.5 for incubation. At designated time points, the supernatant of the mixture (1 ml) was collected and supplemented with fresh PBS (1 ml) before measurement of the absorbance. The release percentage of uPA was determined by the chromogenic substrate method [45]. In the platelet-involved uPA release tests, platelets-rich plasma (200 μl) placed in collagen-coated 96-well plates was set as the negative control, and platelets-rich plasma (180 μl) added with thrombin (1 μM, 20 μl) in the same 96-well plates was set as the positive control.

### Assessment of fibrinolysis kinetic using UV-vis spectrophotometry

In a 96-well plate, fibrinogen (3 mg ml^−1^, 10 l) was incubated for 3 min at 37 °C before thrombin (140 U ml^−1^, 0.25 l) was added. Subsequently, PBS (10 μl), free uPA (1,000 U ml^−1^), and Pkr(Ca/Pda-uPA)-cRGD at pH 7.4, at pH 7.4 with AP (pH 7.4 + AP), at pH 6.5, and at pH 6.5 with AP (pH 6.5 + AP) were added to the mixture right before the measurement of absorbance at the wavelength of 405 nm using a plate reader (BioTek Epoch, USA).The absorbance was continuously recorded for total 240 min. Three replicates were performed for each sample. A blank control was set using 200 μl of PBS.

### Evaluation of static fibrinolysis and thrombolysis

The fibrinolytic performance and thrombolytic capacity were assessed by an agar plate model and a halo blood clot model [49]. PBS at pH 7.4, at pH 7.4 with AP (pH 7.4 + AP), at pH 6.5, and at pH 6.5 with AP (pH 6.5 + AP) were set as assay groups to evaluate pH-responsive and platelets-responsive properties.

### Microfluidic system for evaluation of dynamic targeted thrombolysis

A mixture of ADP (50 μg ml^−1^) and collagen-I solution (0.5 mg ml^−1^) coated Vena8 Fluoro^+^ biochip (2.8 cm by 0.4 mm by 0.1 mm; Cellix Ltd., Ireland) overnight at 4 °C. Then, mouse blood was preincubated with DIOC6 for 3 min and perfused into collagen-coated channels at a wall shear rate of 1,000 s^−1^ at 25 °C for 10 min to generate stable nonocclusive thrombi. Next, blood containing Pkr(Ca/Pda)-cRGD, Pkr(Ca/Pda-uPA)-cRGD, or free uPA was perfused into these channels at a wall shear rate of 1,000 s^−1^. Fluorescence images of thrombi were then acquired to monitor the elimination of thrombi.

### Study on in vivo biodistribution of uPA

KM mice were injected with Cy5.5-labeled Pkr(Ca/Pda-uPA)-cRGD and Cy5.5-labeled free uPA (equivalent to a dose of 10 mg kg^−1^ of uPA) via tail vein. After 1.5 or 24 h, the mice were euthanized, and the main organs (heart, liver, spleen, lung, and kidney) were harvested for biodistribution studies using the in vivo imaging system (IVIS Lumina Series III).

### Cytotoxicity assay

The HUVECs were utilized to assess the cytotoxicity of CaCO_3_-PDA, Pkr(IR-Ca/Pda), and Pkr(IR-Ca/Pda)-cRGD, which were coincubated in a 96-well plate with 200 μl of cell suspension (10^4^ cells/well). The cytotoxic effects were assessed with the Cell Counting Kit-8. Additionally, the impact of materials on HUVECs was assessed utilizing a live/dead (Calcein AM/PI) cell double staining kit.

### Blood compatibility assay

Hemocompatibility was evaluated according to a previous study [50]. The concentration of Pkr(IR-Ca/Pda-uPA)-cRGD in each group was 0.25, 0.5, 1, 2, and 4 mg ml^−1^. To further evaluate the blood compatibility, blood biochemical indexes, blood routine indexes, and immune response (IgG and IgM) were tested. The rats were randomly divided into 2 groups (3 rats per group). Blood samples (300 μl per rat) were collected from rat hearts for assays.

### Tail bleeding risk assay

After 2 h of intravenous injection with PBS buffer, Pkr(IR-Ca/Pda-uPA)-cRGD and free uPA (equivalent to a dose of 10 mg kg^−1^ of uPA), KM mice were anesthetized for bleeding risk assay [51].

### Pharmacokinetics assay

Sprague-Dawley rats were was injected with FITC-labeled free uPA and FITC-labeled Pkr(IR-Ca/Pda-uPA)-cRGD (equivalent to a dose of 10 mg kg^**−1**^ of uPA) via tail vein. Then, 0.1 ml of blood was drawn from the heart at different times to determine the concentration and half-life time of uPA in blood [52].

### Mouse tail thrombosis model

KM mice with tails longer than 10 cm were starved for 8 h and then injected intraperitoneally with 1% w/v fresh carrageenan saline (20 mg kg^**−1**^) and kept at 20 °C. Black tail thrombosis was seen after about 24 h.

### Evaluation of drug carriers’ penetration to thrombi

Tails of KM mice with thrombi were sectioned and coincubated with FITC-labeled free uPA, Pkr(Ca/Pda-uPA), and Pkr(Ca/Pda-uPA)-cRGD (equivalent to a dose of 2.5 μg ml^**−1**^ of uPA) for 35 min at 37 °C. After rinsed with PBS, the sections were observed with the fluorescence microscope (Eclipse Ni-E, Japan).

### Mouse tail thrombolysis

KM mice with same length of black tail thrombi were randomly divided into 5 groups and injected intravenously with PBS, Pkr(Ca/Pda-uPA), Pkr(Ca/Pda-uPA)-cRGD, and free uPA (equivalent to a dose of 10 mg kg^−1^ of uPA). The length of the caudal thrombi was measured before and after each treatment. After 7 d of observation, all mice were sacrificed to collected tails for histological analysis.

### DVT model

The abdominal cavity of Sprague-Dawley rats was opened by a 3-cm incision in the middle of the abdominal wall. The whole intestine was then pulled aside and covered by a gauze saturated with saline. Next, a 4-0 suture was placed along with the IVC before a 5-0 suture was used to tie the IVC and 4-0 suture together firmly near the left renal vein at 5 mm. The 4-0 suture was then drawn away from the IVC to leave a stenosis of IVC. In addition, all visible collateral branches between the left renal vein and the iliolumbar vein were ligated with 5-0 suture. Finally, the DVT model was established 12 h after the stenosis of IVC. All sutures tied on vessels were removed before thrombolytic therapies, allowing for drug delivery to thrombi of DVT.

### In vivo real-time NIR-II FLI for DVT

The real-time NIR-II FLI of thrombi of DVT was performed right before the injection of Pkr(IR-Ca/Pda-uPA)-cRGD or Pkr(IR-Ca/Pda)-cRGD through the tail vein of rats. Thrombus images of DVT were acquired at different time intervals to record the evolution of thrombi during thrombolysis over time. In the therapeutic scenario using Pkr(IR-Ca/Pda-uPA)-cRGD, all vessels were collected after thrombolytic therapy and stained with hematoxylin and eosin for histological analysis.

### Statistical analysis

Data were presented as mean ± standard deviation (SD). Statistical analyses were performed using software GraphPad version 8.0 Prism. *P* values were calculated using the Student *t* test (for comparison) or 1-way analysis of variance (ANOVA), and all error bars depicted the mean ± standard deviation of at least 3 independent experiments, unless otherwise stated. Statistical significance was assessed at *P <0.05, **P < 0.01, ***P < 0.001, and ****P < 0.0001.

## Data Availability

All data needed to evaluate the conclusions is present in the paper and the supplementary materials. Additional data related to the paper may be requested from the authors.
