# Supplementary material for: pH-Responsive Theranostic Colloidosome Drug Carriers Enable Real-Time Imaging of Targeted Thrombolytic Process with Near-Infrared-II for Deep Venous Thrombosis
Source: Research (Wash D C). 2024 May 29;7:0388. doi: 10.34133/research.0388 (PMC11136571; doi:10.34133/research.0388)
Supplement: Supplementary 1 — Figs. S1 to S19 [file research.0388.f1.zip › SI_30 Apr.docx]

**Supporting Information**

**Title**

pH-Responsive Theranostic Colloidosome Drug Carriers Enable Real-Time Imaging of Targeted Thrombolytic Process with Near-Infrared-II for Deep Venous Thrombosis

**Authors**

Yaxin Ye^1^, Zhechang Chen^1^, Shengzhang Zhang^2^, Paul Slezak^3^, Fei Lu^1^, Ruiqi Xie^1,3^, Dongwon Lee^4^†, Guangqian Lan^1^†, Enling Hu^5^†

**Affiliations**

^1^ State Key Laboratory of Resource Insects, College of Sericulture, Textile and Biomass Sciences, Southwest University, Chongqing 400715, China.

^2^ Department of Cardiovascular Medicine, Yueqing People's Hospital, Wenzhou 325699, China.

^3^ Ludwig Boltzmann Institute for Experimental and Clinical Traumatology, AUVA Research Center, 1200 Vienna, Austria.

^4^ Department of Bionanotechnology and Bioconvergence Engineering and Department of Polymer·Nano Science and Technology, Jeonbuk National University, Jeonju, Chonbuk 54896, Republic of Korea.

^5^ School of Fashion and Textiles, The Hong Kong Polytechnic University, Hong Kong.

† Corresponding author at: School of Fashion and Textiles, The Hong Kong Polytechnic University, Kowloon, Hong Kong.

E-mail addresses: [enling.allen.hu@connect.polyu.hk](mailto:enling.allen.hu@connect.polyu.hk) (E.L. Hu), [j070218@swu.edu.cn](mailto:j070218@swu.edu.cn) (G.Q. Lan) and [dlee@jbnu.ac.kr](mailto:dlee@jbnu.ac.kr) (D.W. Lee)


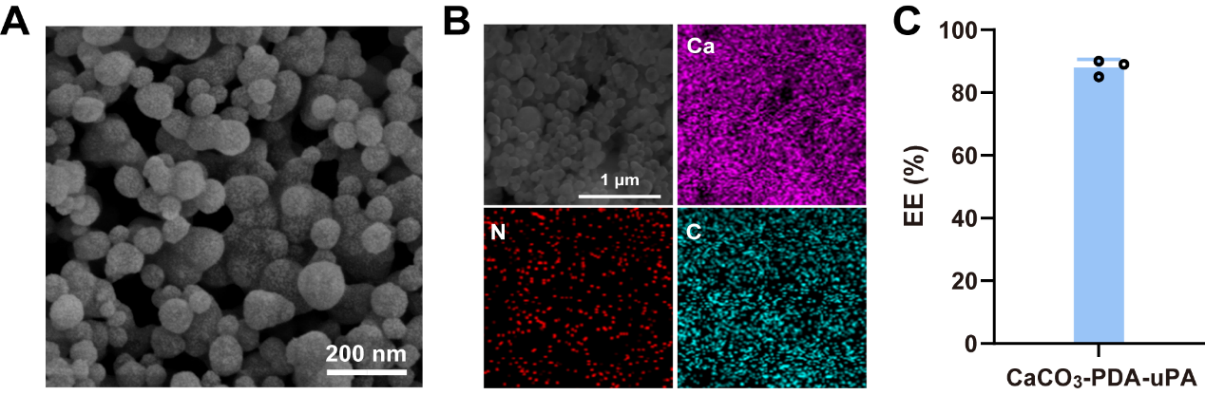


**Figure S1.** (A) Scanning electron microscopy (SEM) images of CaCO_3_-PDA hollow nanoparticles. (B) CaCO_3_-PDA hollow nanoparticles was subjected to mapping analysis showing the distribution of N, C and Ca. (C) The uPA encapsulation efficiency (EE%) of CaCO_3_-PDA-uPA.


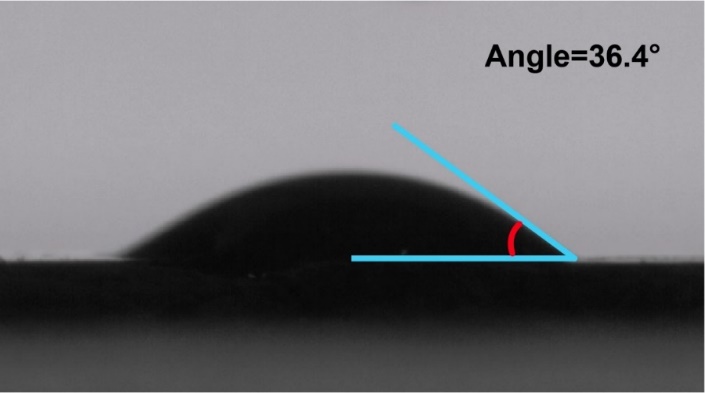


**Figure S2.** Static water contact angle indicating CaCO_3_-PDA-uPA hydrophilic properties.


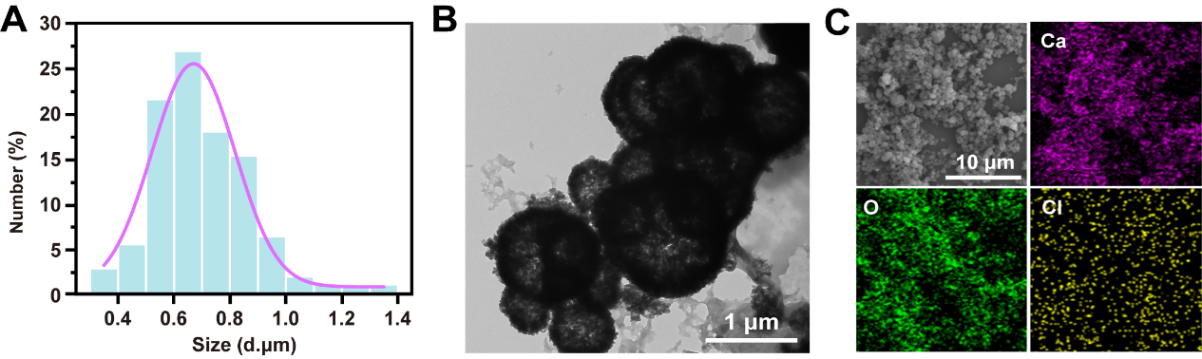


**Figure S3.** (A) Statistical plot of the particle size distribution of Pkr(Ca/Pda-uPA). (B) SEM images of Pkr(IR-Ca/Pda-uPA) particles. (C) Pkr(IR-Ca/Pda-uPA) was subjected to mapping analysis showing the distribution of Cl, O and Ca in the synthesized Pkr(IR-Ca/Pda-uPA) self-assembled particles.


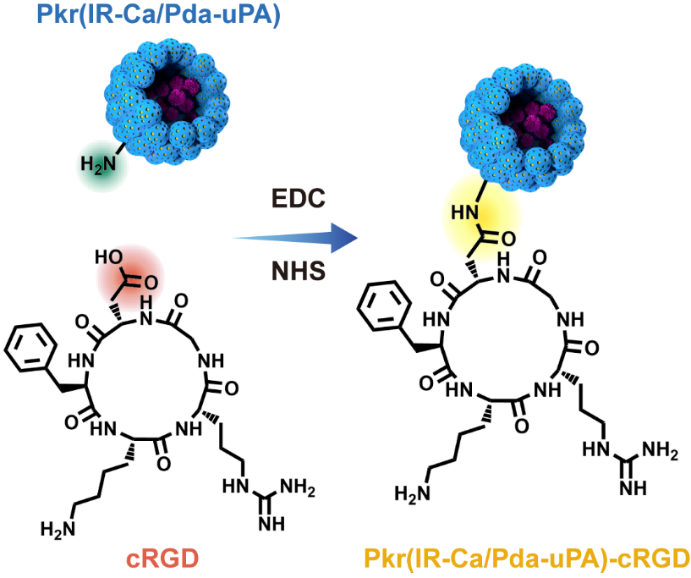


**Figure S4.** Synthesis of Pkr(IR-Ca/Pda-uPA)-cRGD conjugate from Pkr(IR-Ca/Pda-uPA) and cRGD peptides.


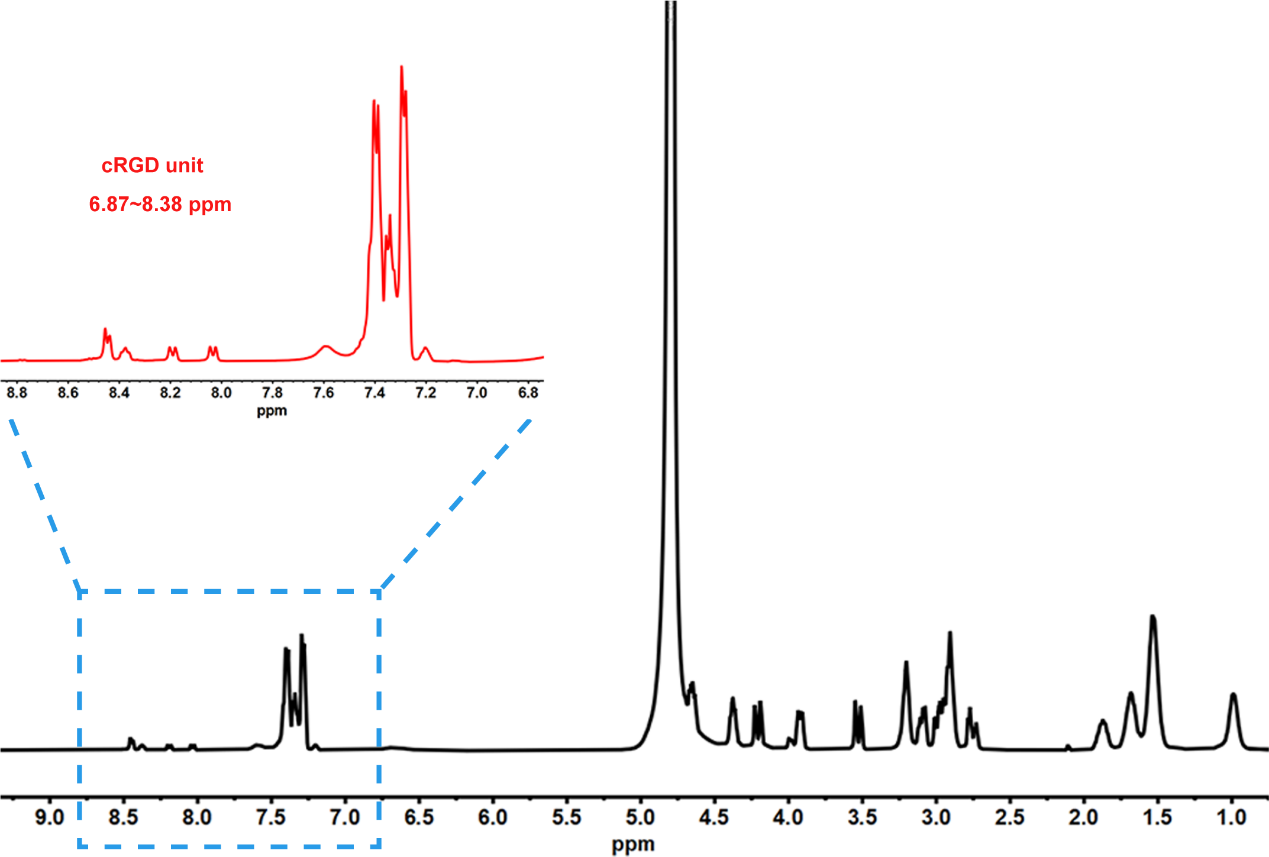


**Figure S5.** ^1^H NMR spectrum of Pkr(Ca/Pda)-cRGD in D_2_O (400 MHz, 25 °C).


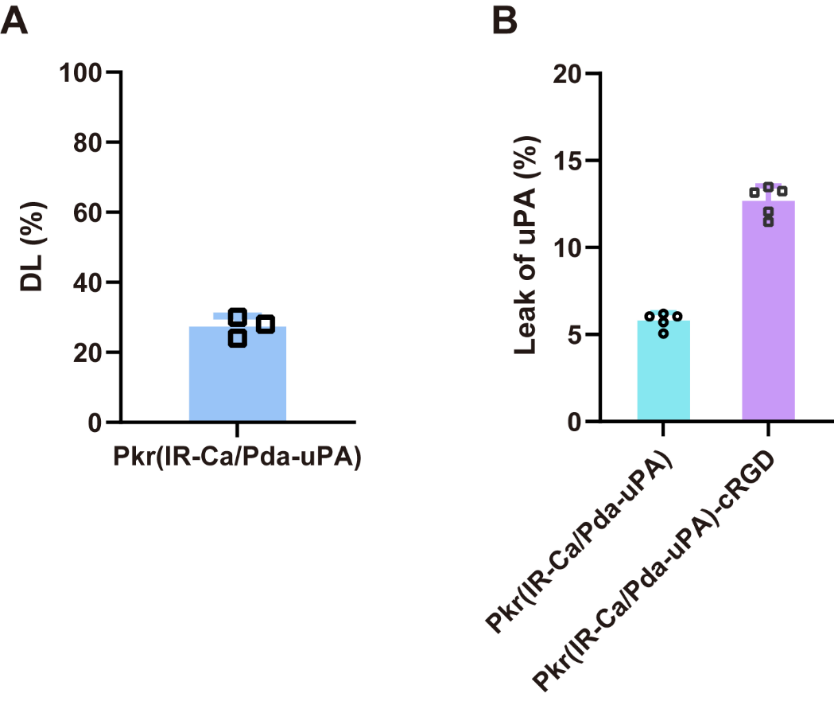


**Figure S6.** (A) The IR-1048 loading capacity (DL%) in Pkr(IR-Ca/Pda-uPA). (B) Leakage rate of uPA during the assembly of CaCO_3_-PDA for preparation of Pkr(IR-Ca/Pda-uPA) and grafting of cRGD onto Pkr(IR-Ca/Pda-uPA) for preparation of Pkr(IR-Ca/Pda-uPA)-cRGD.


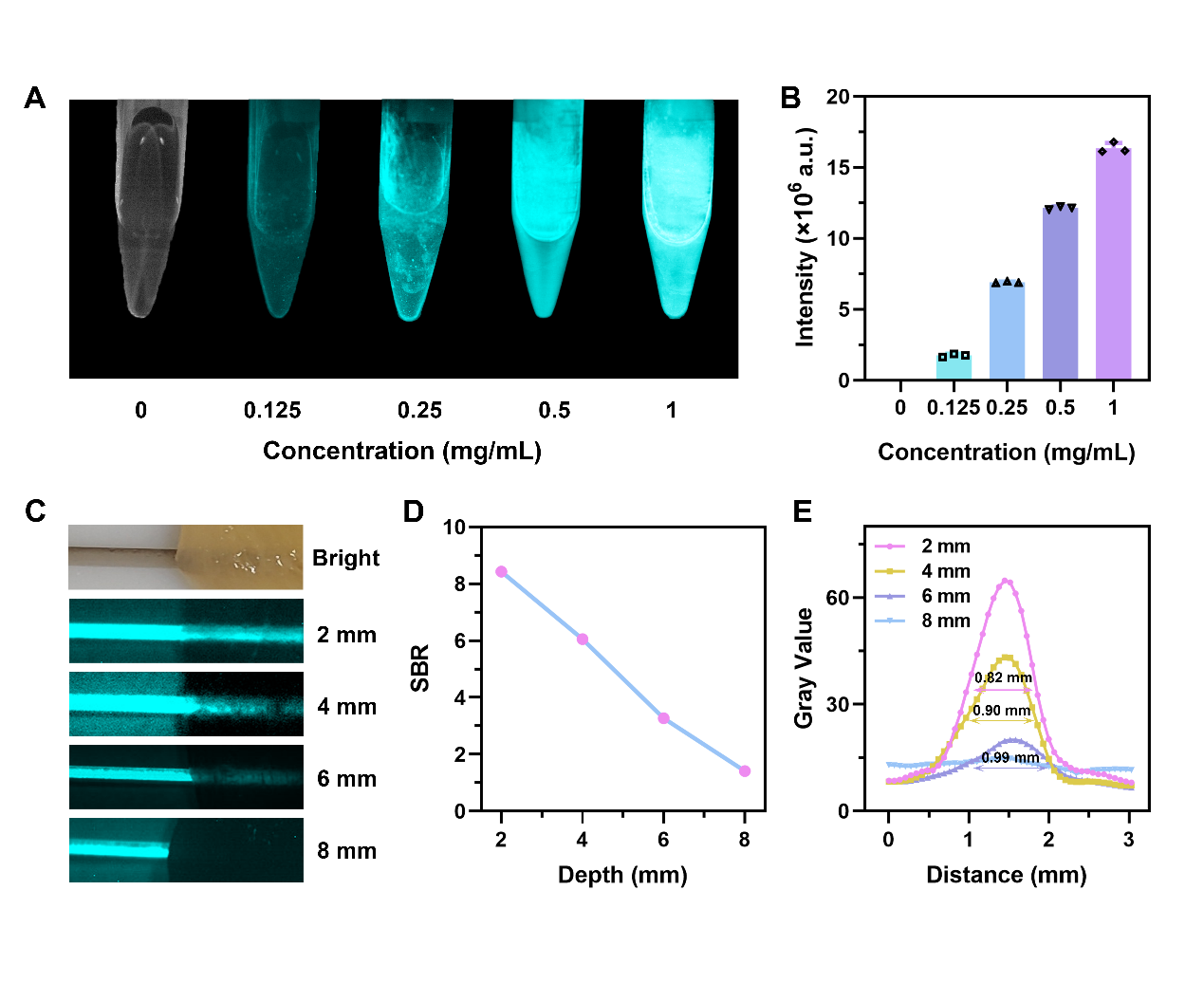


**Figure S7.** **NIR-II imaging capacity and tissue penetration depth of Pkr(IR-Ca/Pda-uPA)-cRGD.** (A) NIR-II images of Pkr(IR-Ca/Pda-uPA)-cRGD at 1064 nm. (B) Fluorescence signal intensity. (C) Penetration depth under different thickness of chicken breasts. (D) Signal-to-noise ratio. (E) Full width at half maxima.


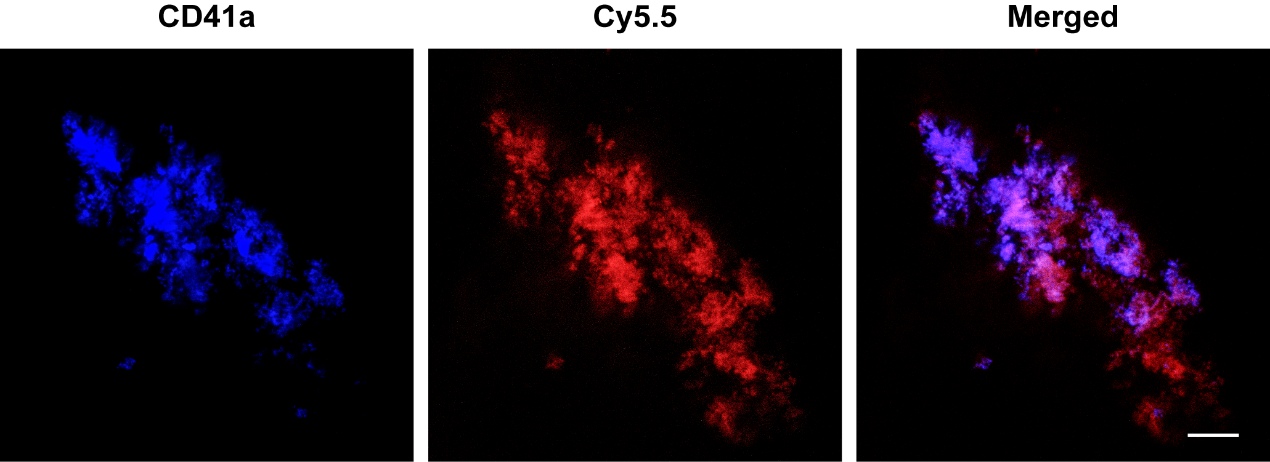


Figure S8. CLSM images of the co-localization of cRGD peptides from Pkr(Ca/Pda-uPA)-cRGD and α_IIb_β_3_ integrins from activated platelets (scale bar: 10 μm).


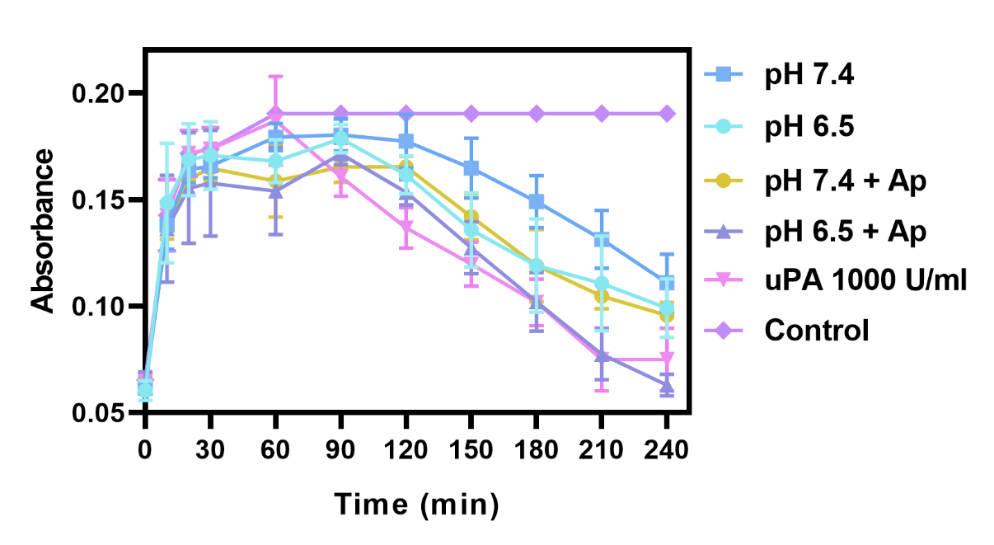


**Figure S9.** Absorbance of incubation mixtures containing fibrinogen, thrombin and/or Pkr(Ca/Pda-uPA)-cRGD.


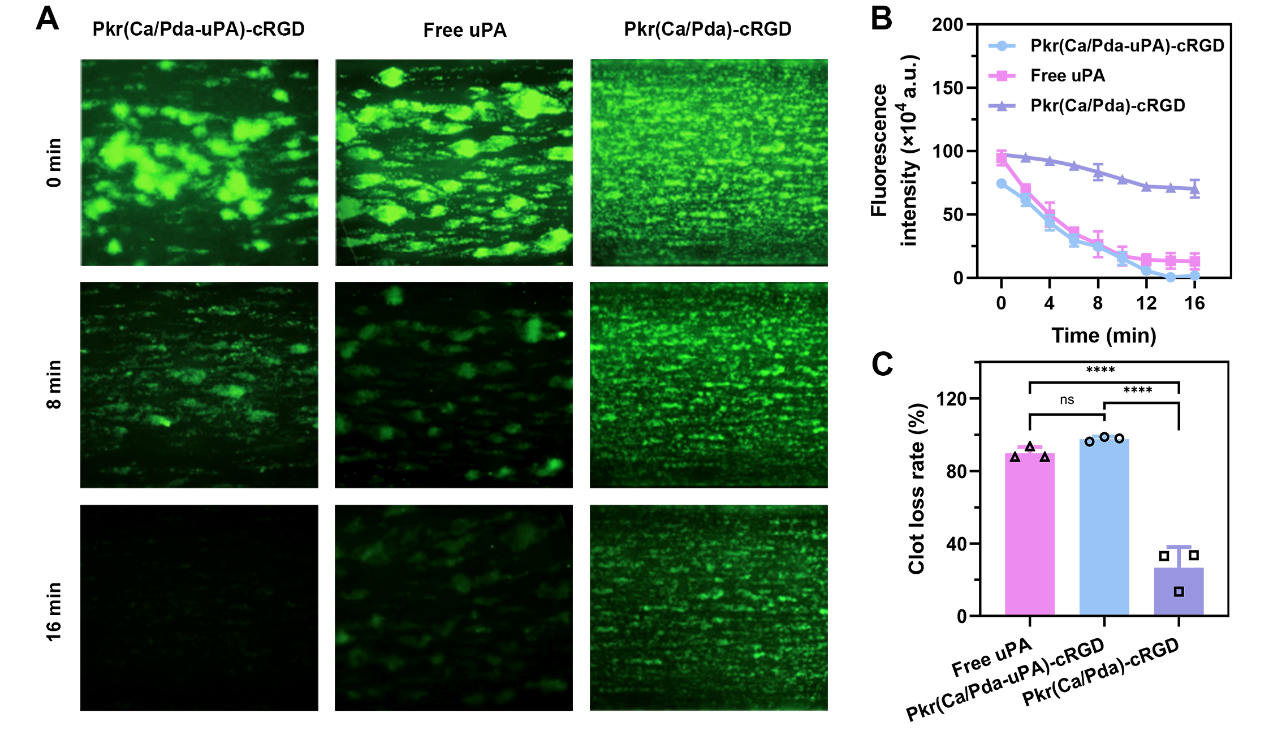


**Figure S10.** **Flow conditions for in vitro targeted thrombolysis.** (A) Representative fluorescence images of thrombi in the flow chamber. (B) Real-time changes of fluorescence at the thrombus. (C) Clot loss rate after 16 min of perfusion.


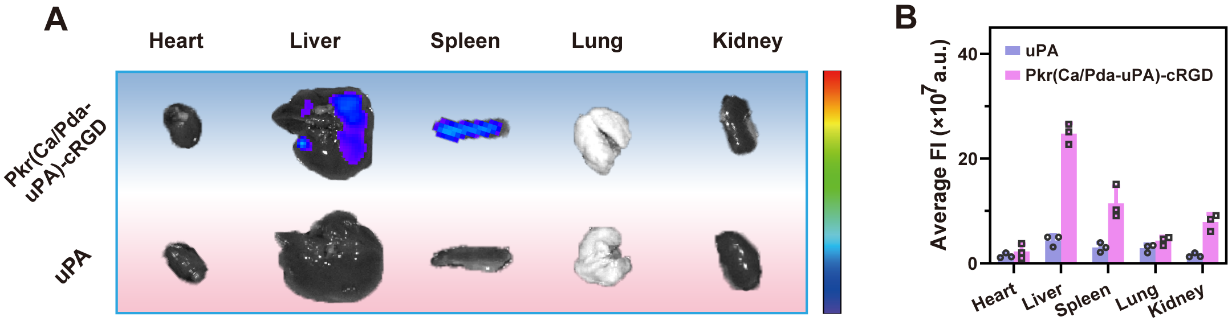


**Figure S11.** (A) FLI of mouse organs after intravenous injection (24 h later). (B) Fluorescence intensity of organs.


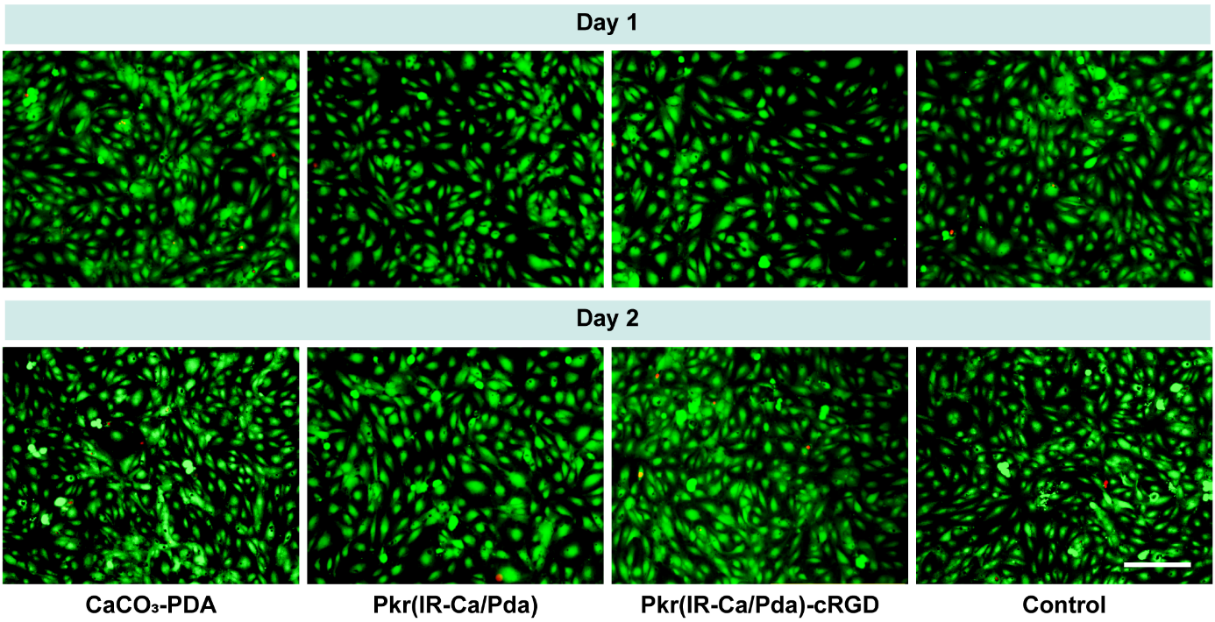


**Figure S12.** Fluorescence microscopy images of human umbilical vein endothelial cells (HUVECs) after co-incubation with CaCO_3_-PDA, Pkr(IR-Ca/Pda), and Pkr(IR-Ca/Pda)-cRGD (scale bar: 100 μm).


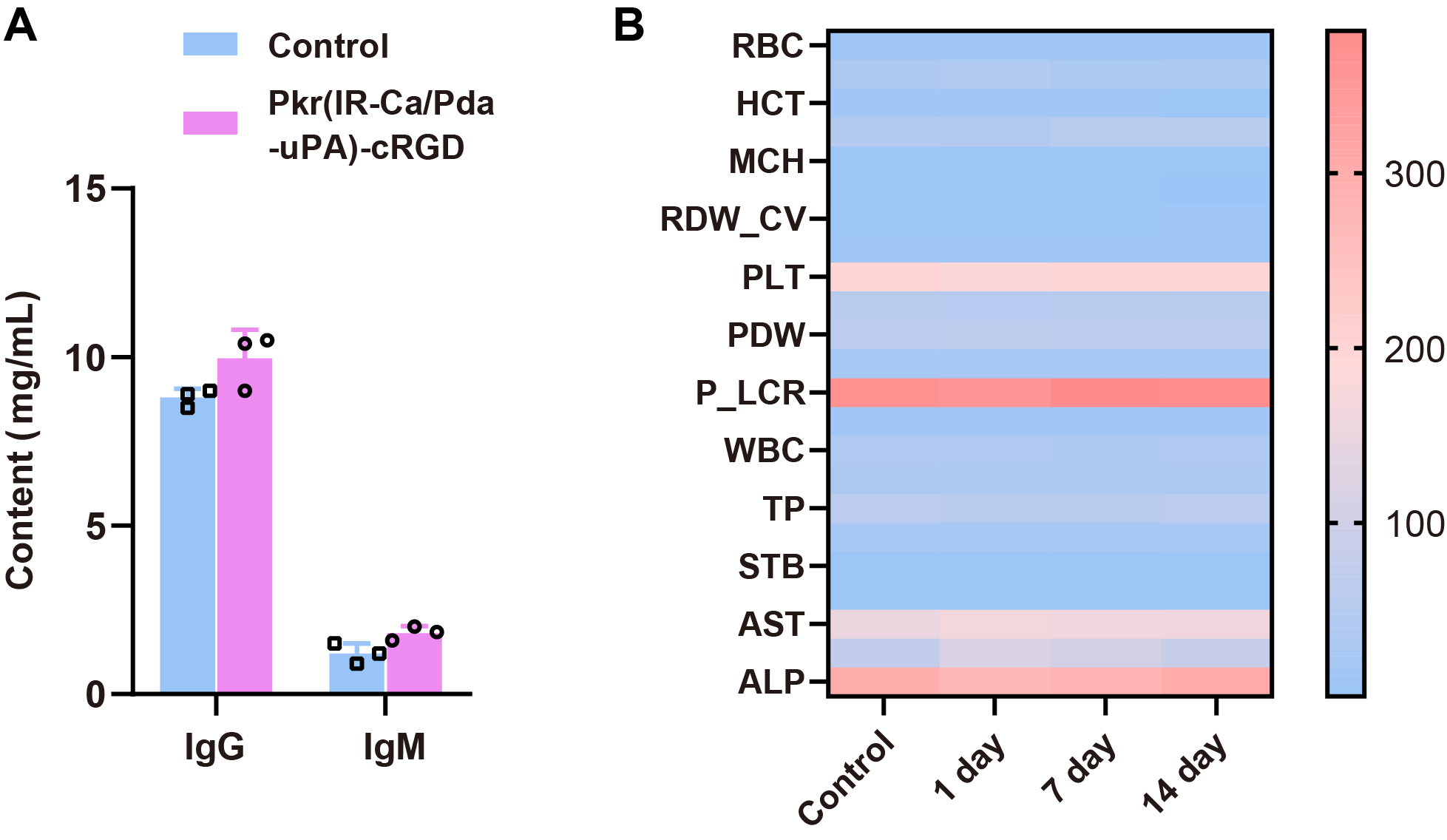


Figure S13. (A) Comparison of immunoglobulin levels at 3 days Pkr(IR-Ca/Pda-uPA)-cRGD injection (B) Blood biochemical indexes and blood routine indexes in rats on days 1, 7 and 14 after injection of Pkr(IR-Ca/Pda-uPA)-cRGD.


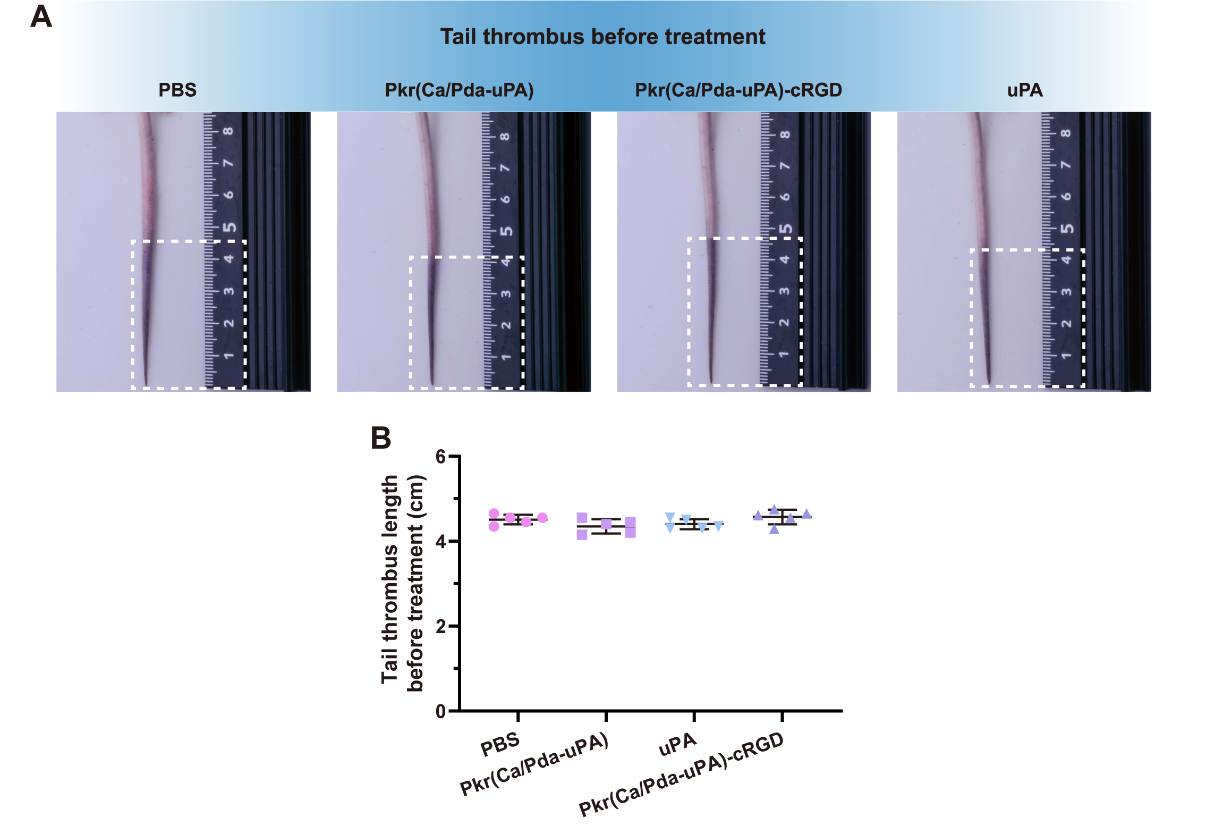


**Figure S14**. (A) Photographs of black tail thrombi used for treatment with PBS, Pkr(Ca/Pda-uPA), Pkr(Ca/Pda-uPA)-cRGD and free uPA. (B) Tail thrombus length of mice before treatment.


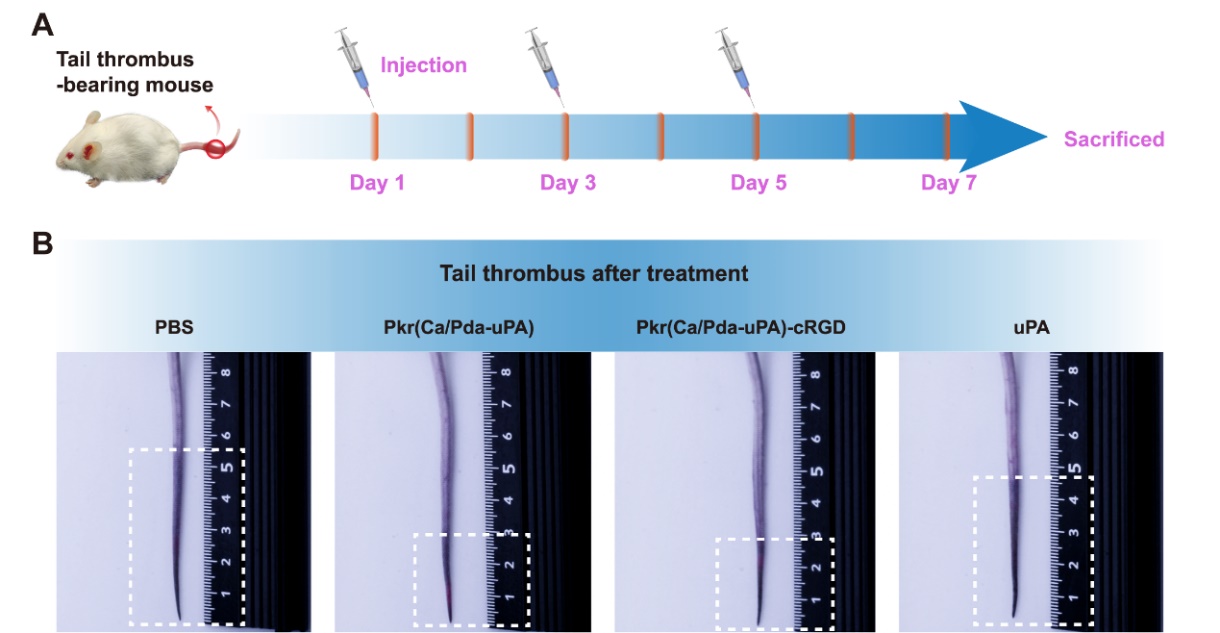


**Figure S15.** (A) Treatment flow for mice with tail thrombus. (B) Tail thrombus length of mice after treatment.


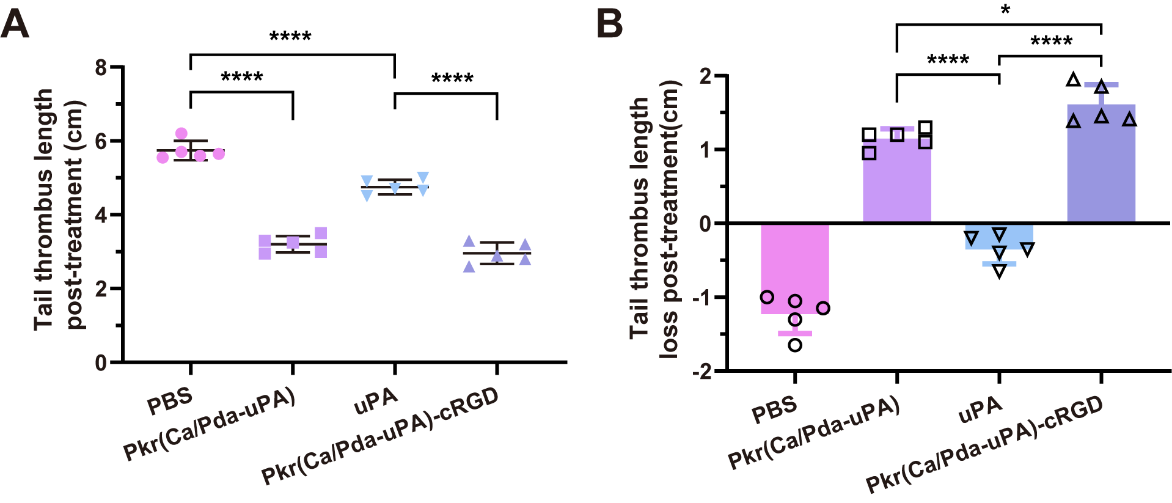


**Figure S16.** (A) Tail thrombus length of mice after treatment. (B) Tail thrombus length loss after treatment.


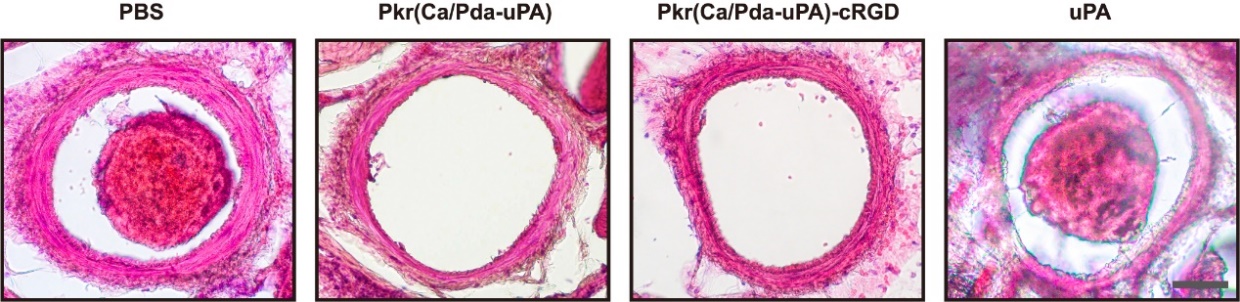


**Figure S17.** H&E-stained tail sections of KM mice after treatment (scale bar: 100 μm).


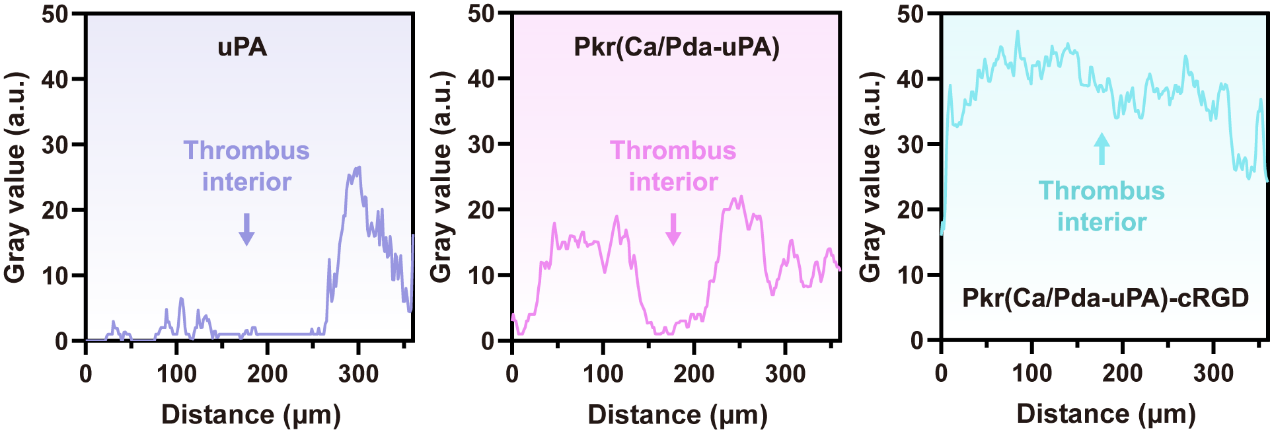


**Figure S18.** Intensity profiles of the cross-sections of tail thrombi (dashed lines in Figure 8(B)) using image J).


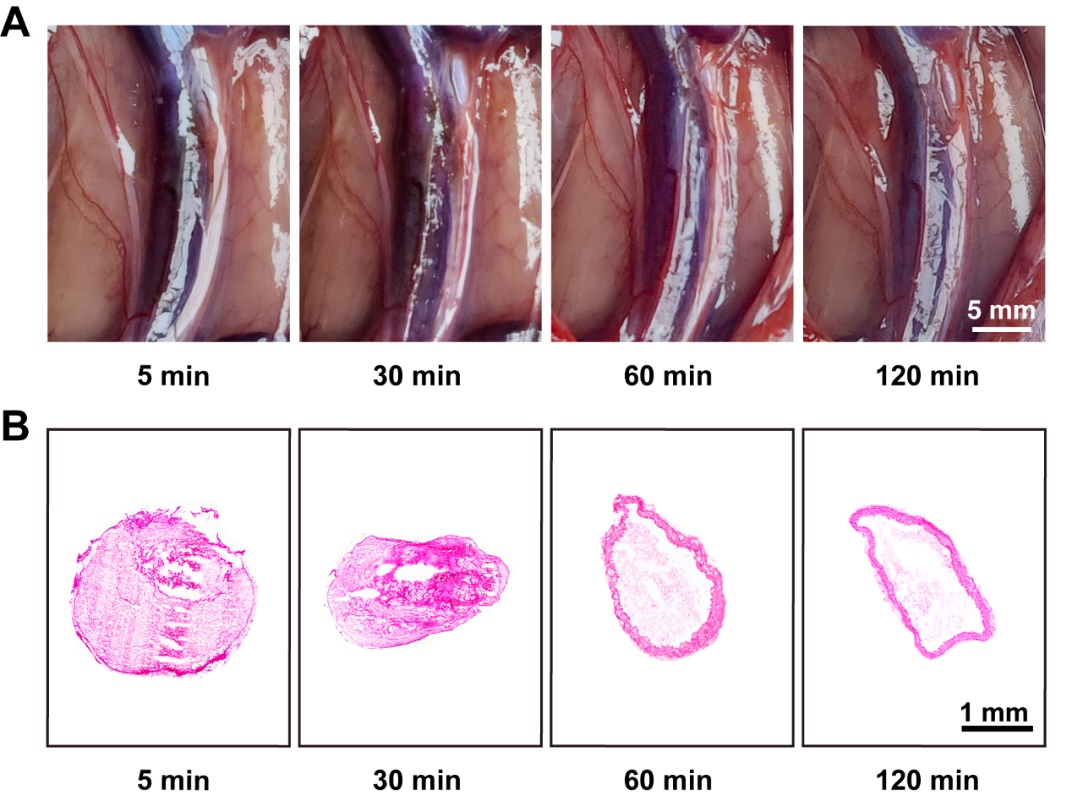


**Figure S19.** (A) Real-time images of representative IVC during thrombolytic therapy. (B) H&E staining of histological sections of IVC with thrombus.
