# Supplementary figures and images for: pH-Responsive Theranostic Colloidosome Drug Carriers Enable Real-Time Imaging of Targeted Thrombolytic Process with Near-Infrared-II for Deep Venous Thrombosis
Source: Research (Wash D C). 2024 May 29;7:0388. doi: 10.34133/research.0388 (PMC11136571; doi:10.34133/research.0388)

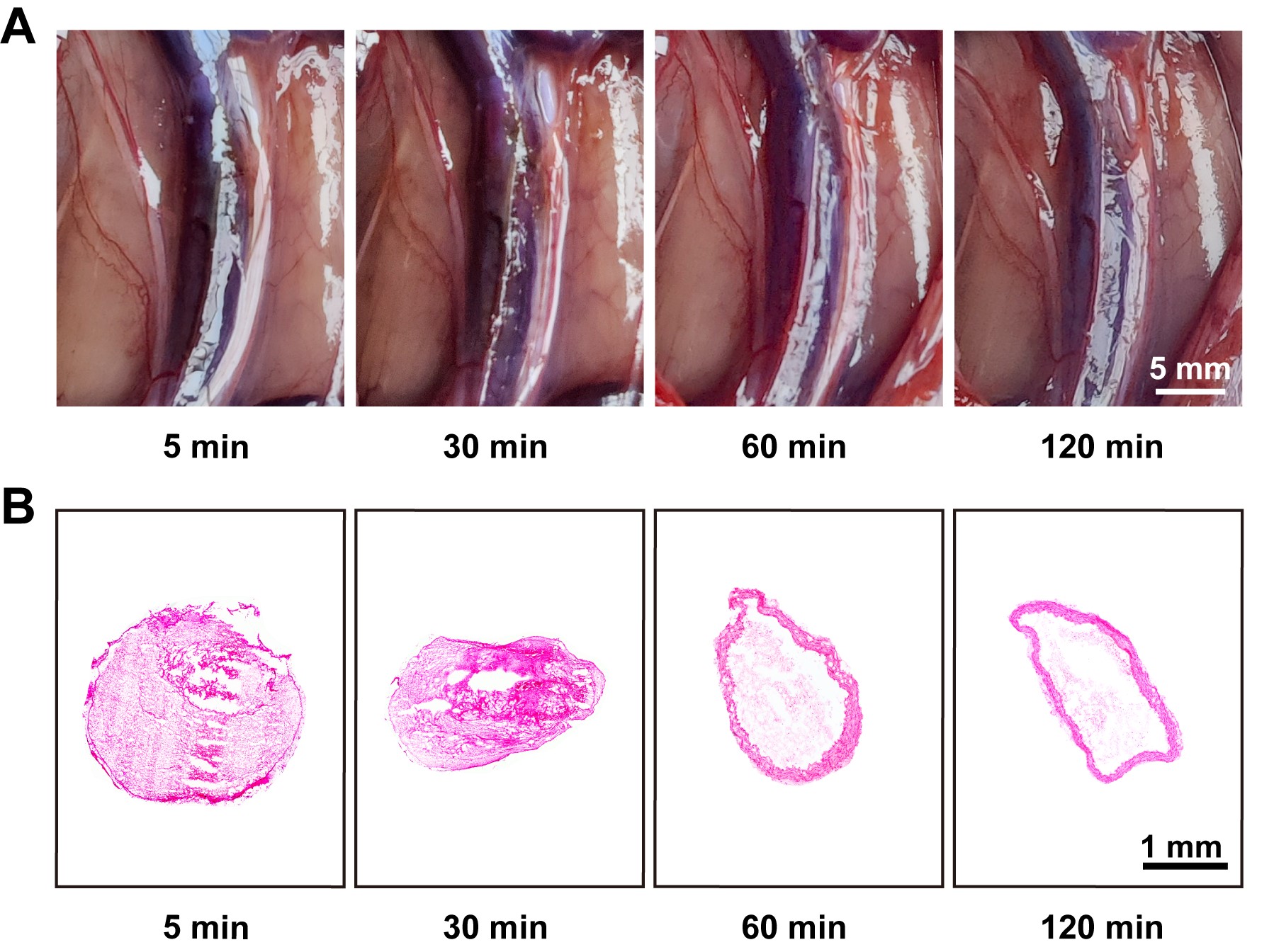

Supplement: Supplementary 1 — Figs. S1 to S19 [file research.0388.f1.zip › S19.jpg]

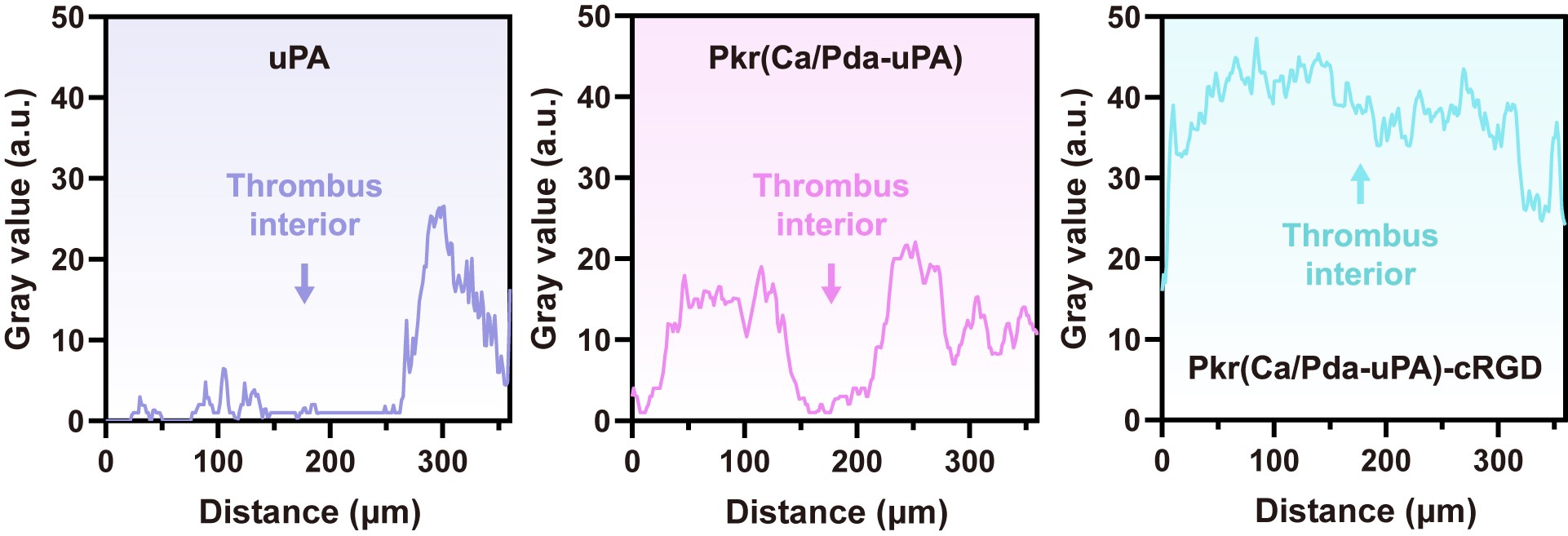

Supplement: Supplementary 1 — Figs. S1 to S19 [file research.0388.f1.zip › S18.jpg]

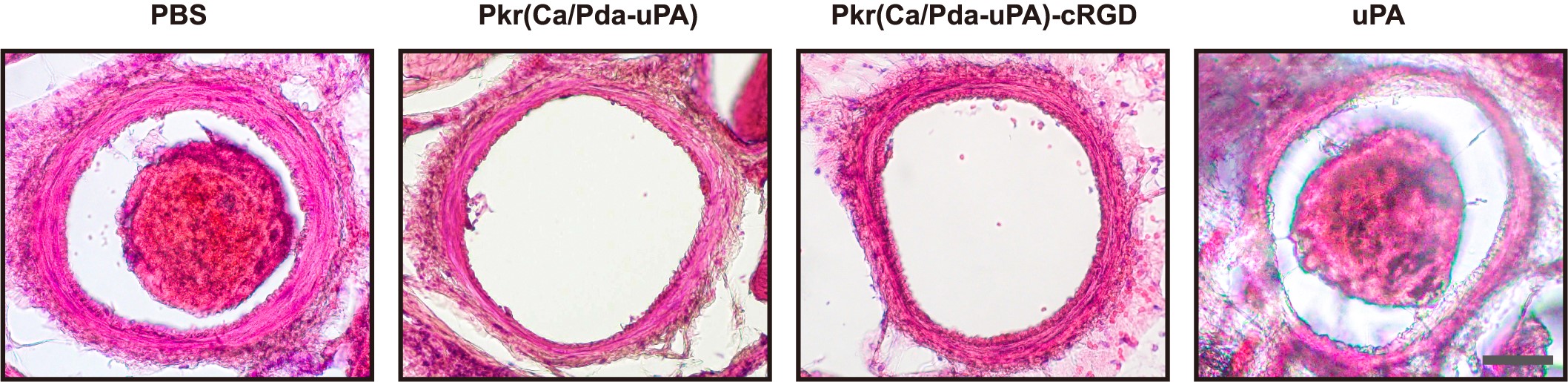

Supplement: Supplementary 1 — Figs. S1 to S19 [file research.0388.f1.zip › S17.jpg]

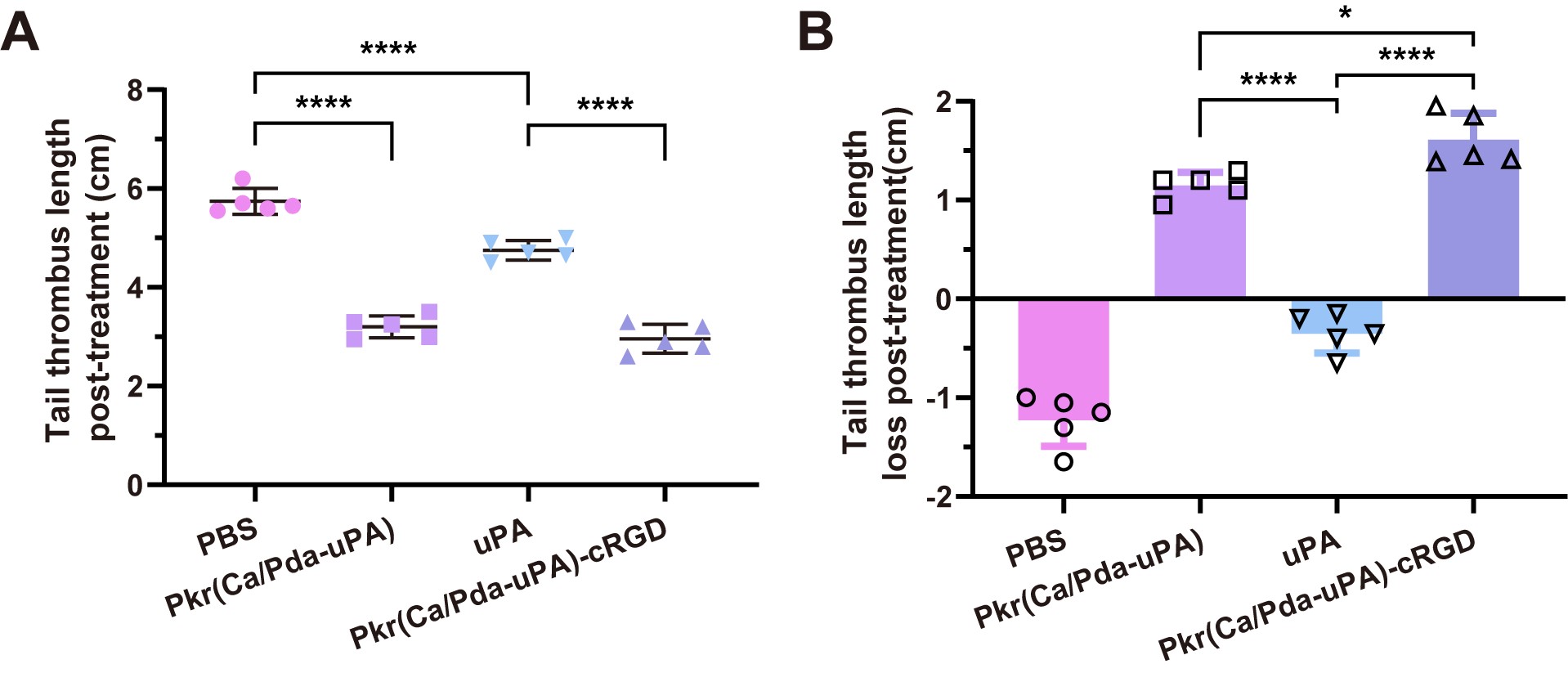

Supplement: Supplementary 1 — Figs. S1 to S19 [file research.0388.f1.zip › S16.jpg]

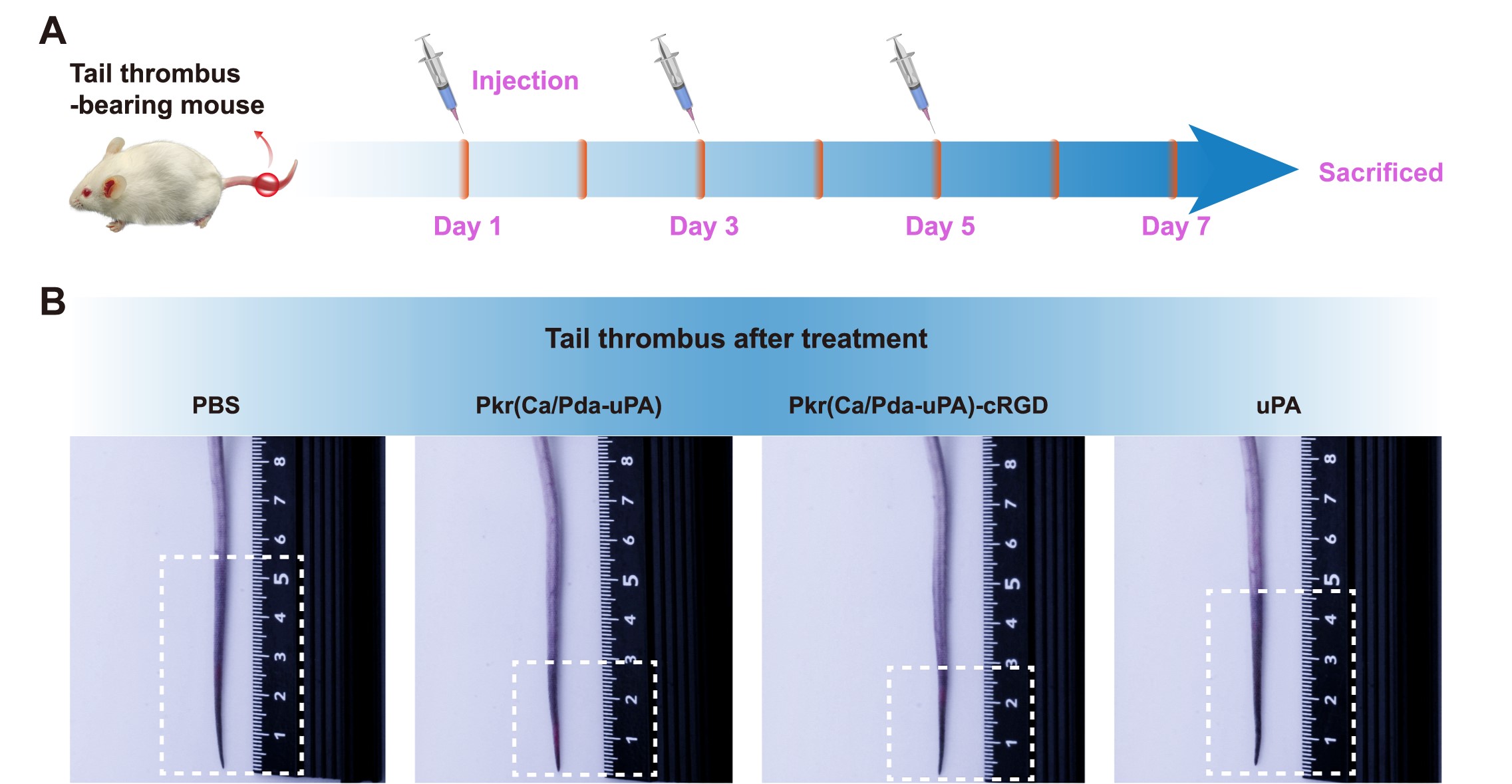

Supplement: Supplementary 1 — Figs. S1 to S19 [file research.0388.f1.zip › S15.jpg]

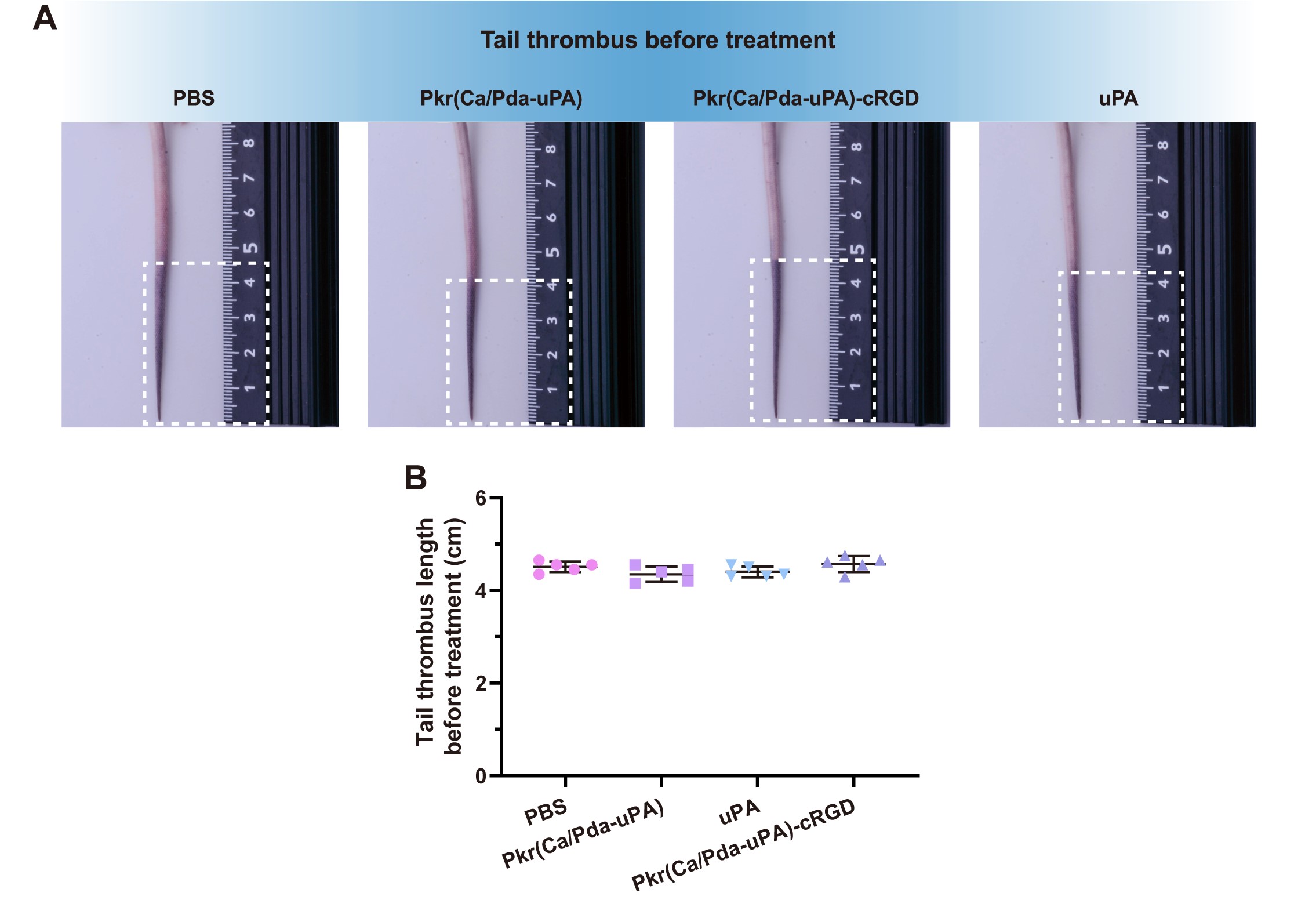

Supplement: Supplementary 1 — Figs. S1 to S19 [file research.0388.f1.zip › S14.jpg]

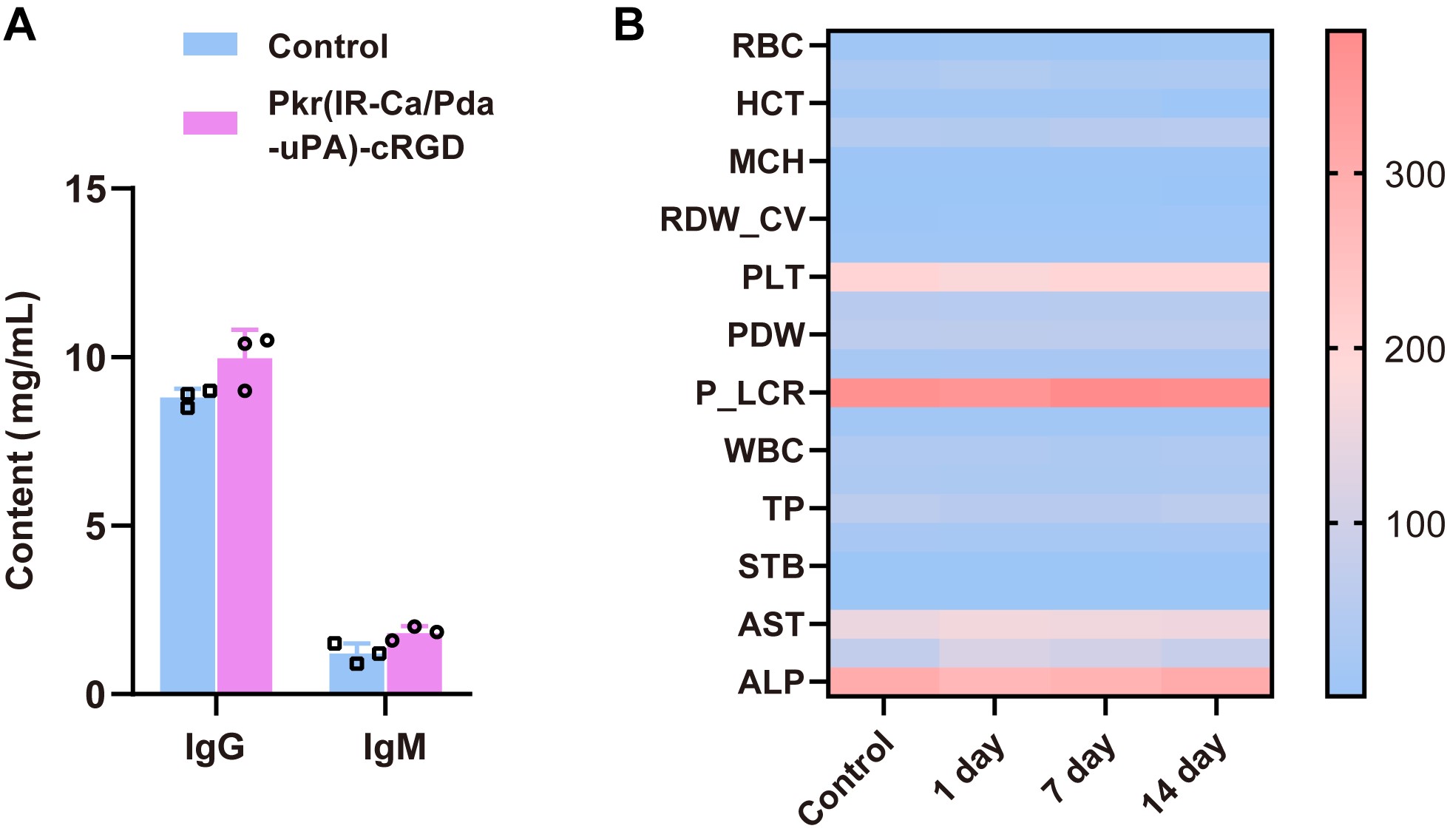

Supplement: Supplementary 1 — Figs. S1 to S19 [file research.0388.f1.zip › S13.jpg]

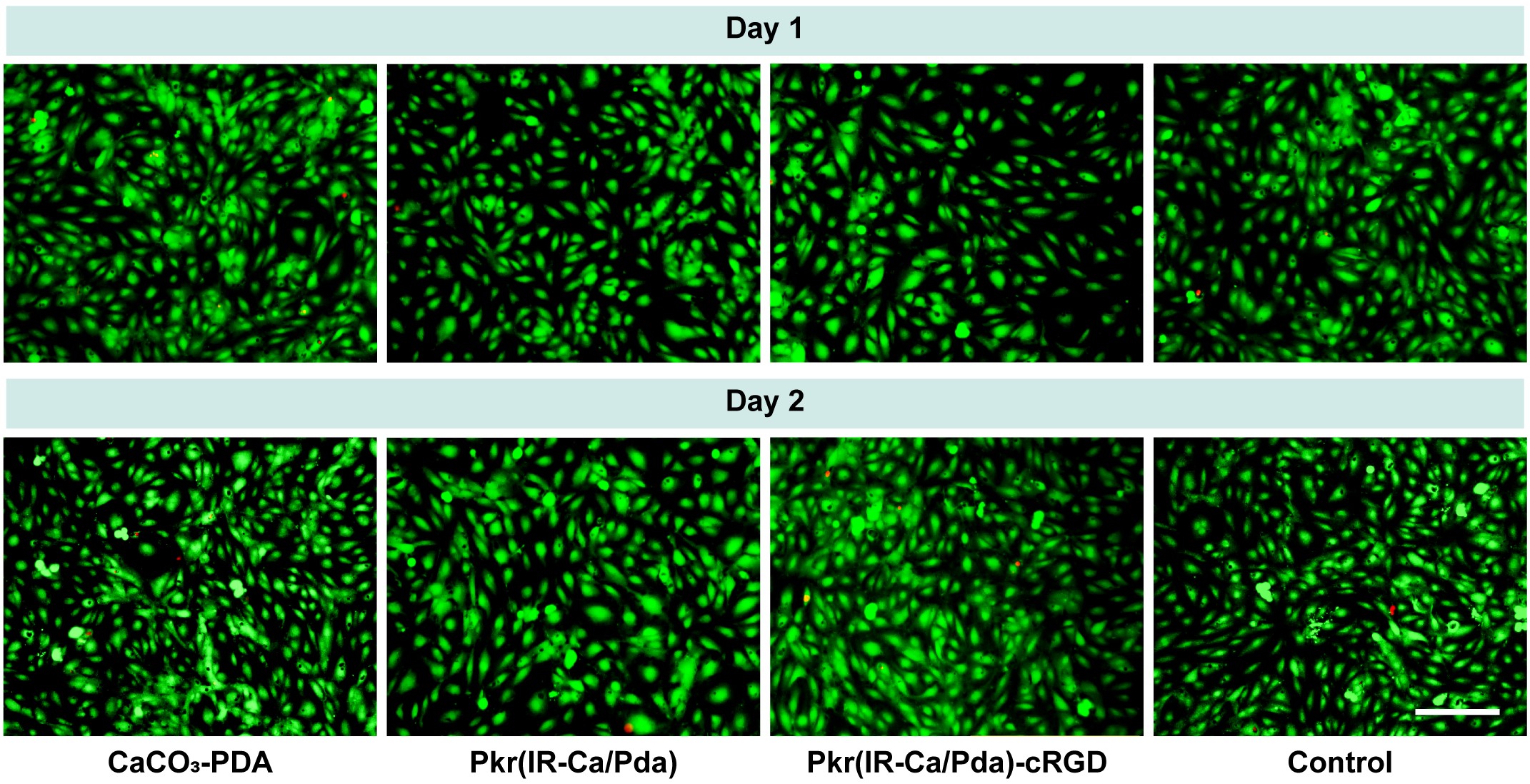

Supplement: Supplementary 1 — Figs. S1 to S19 [file research.0388.f1.zip › S12.jpg]

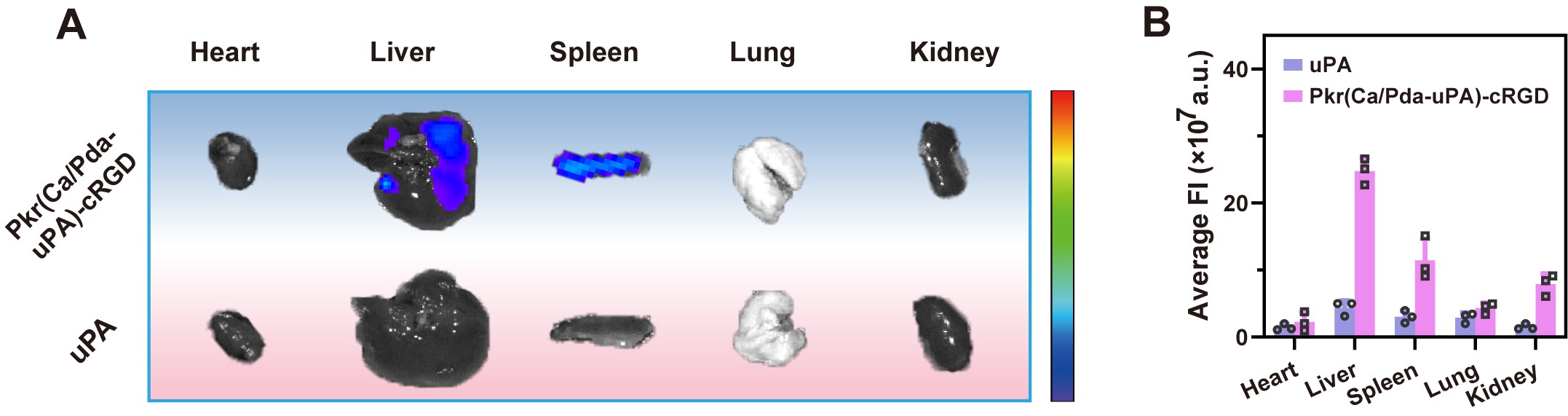

Supplement: Supplementary 1 — Figs. S1 to S19 [file research.0388.f1.zip › S11.jpg]

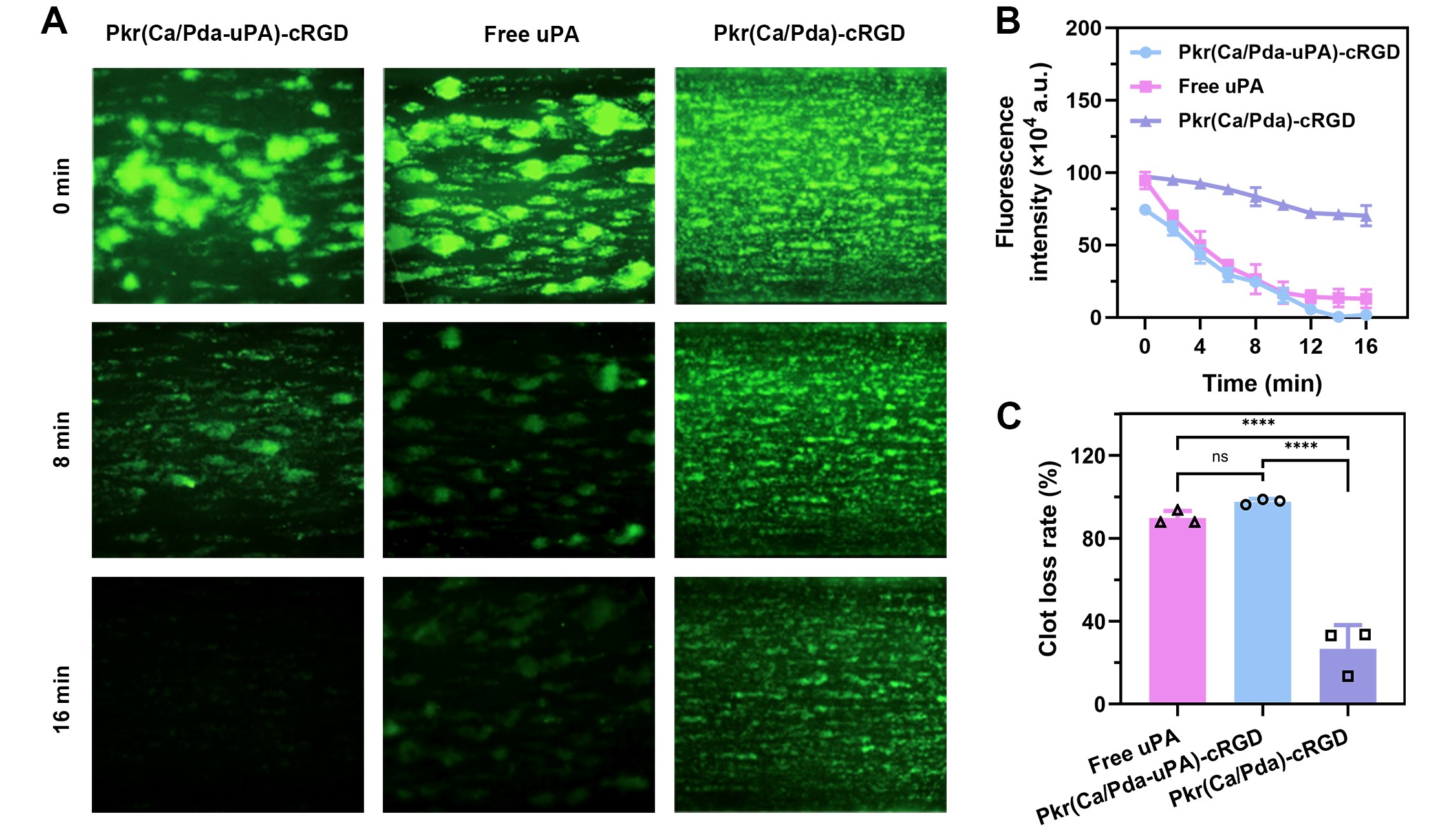

Supplement: Supplementary 1 — Figs. S1 to S19 [file research.0388.f1.zip › S10.jpg]

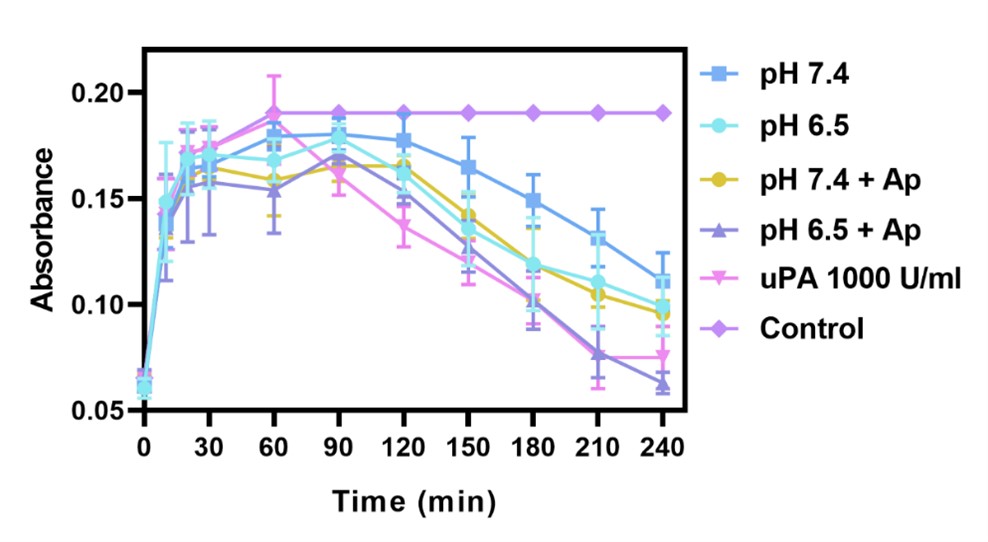

Supplement: Supplementary 1 — Figs. S1 to S19 [file research.0388.f1.zip › S9.jpg]

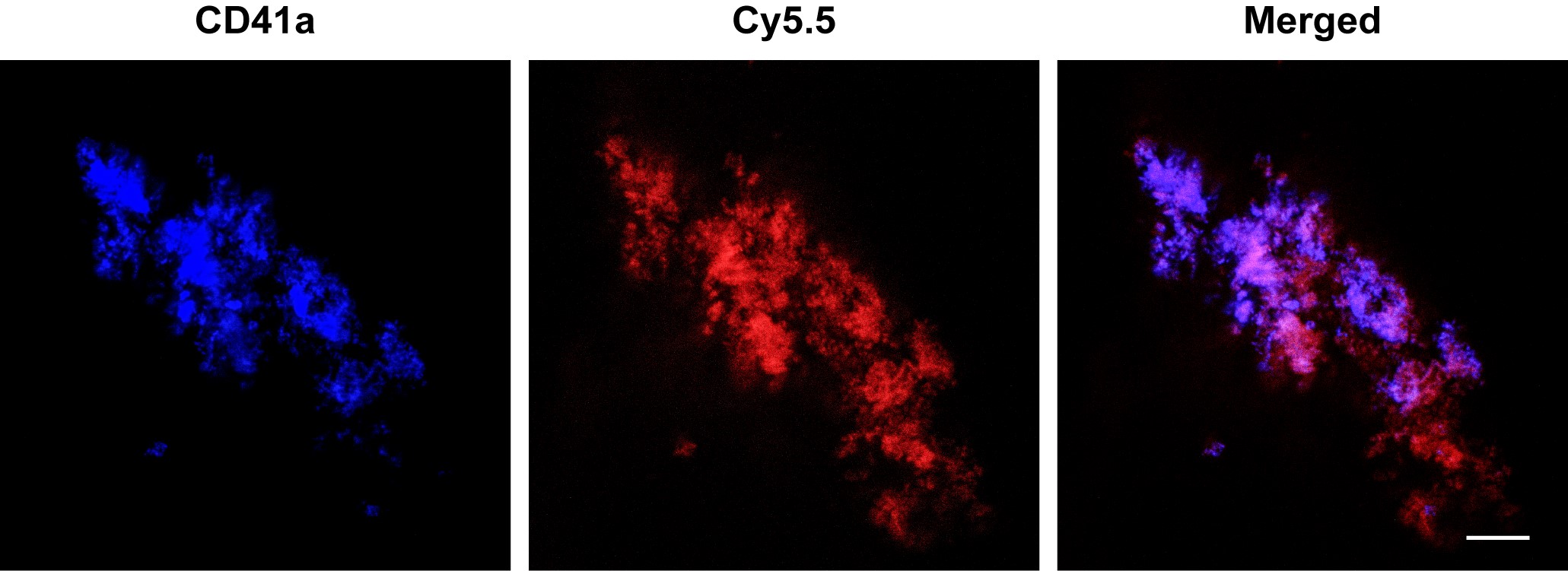

Supplement: Supplementary 1 — Figs. S1 to S19 [file research.0388.f1.zip › S8.jpg]

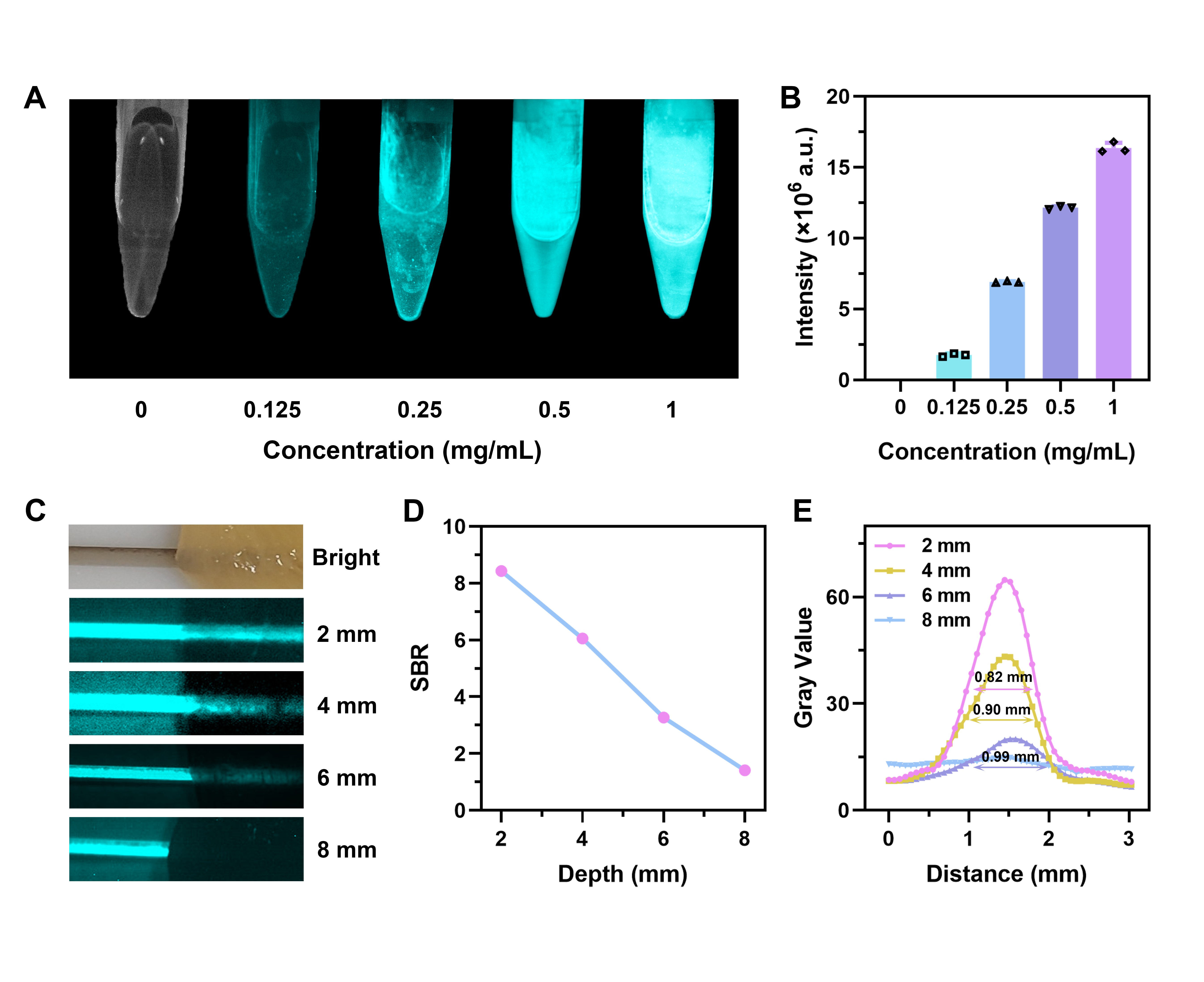

Supplement: Supplementary 1 — Figs. S1 to S19 [file research.0388.f1.zip › S7.jpg]

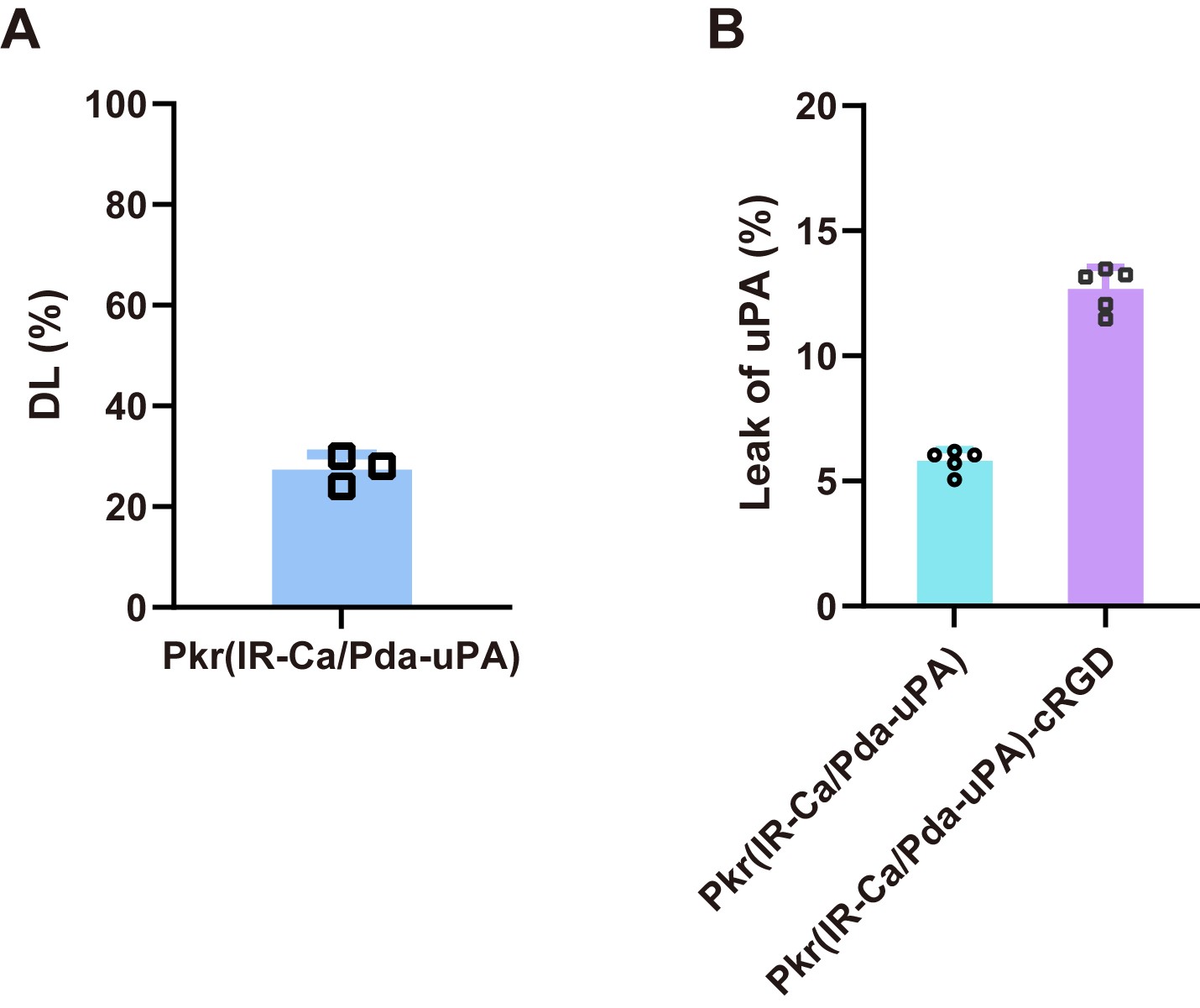

Supplement: Supplementary 1 — Figs. S1 to S19 [file research.0388.f1.zip › S6.jpg]

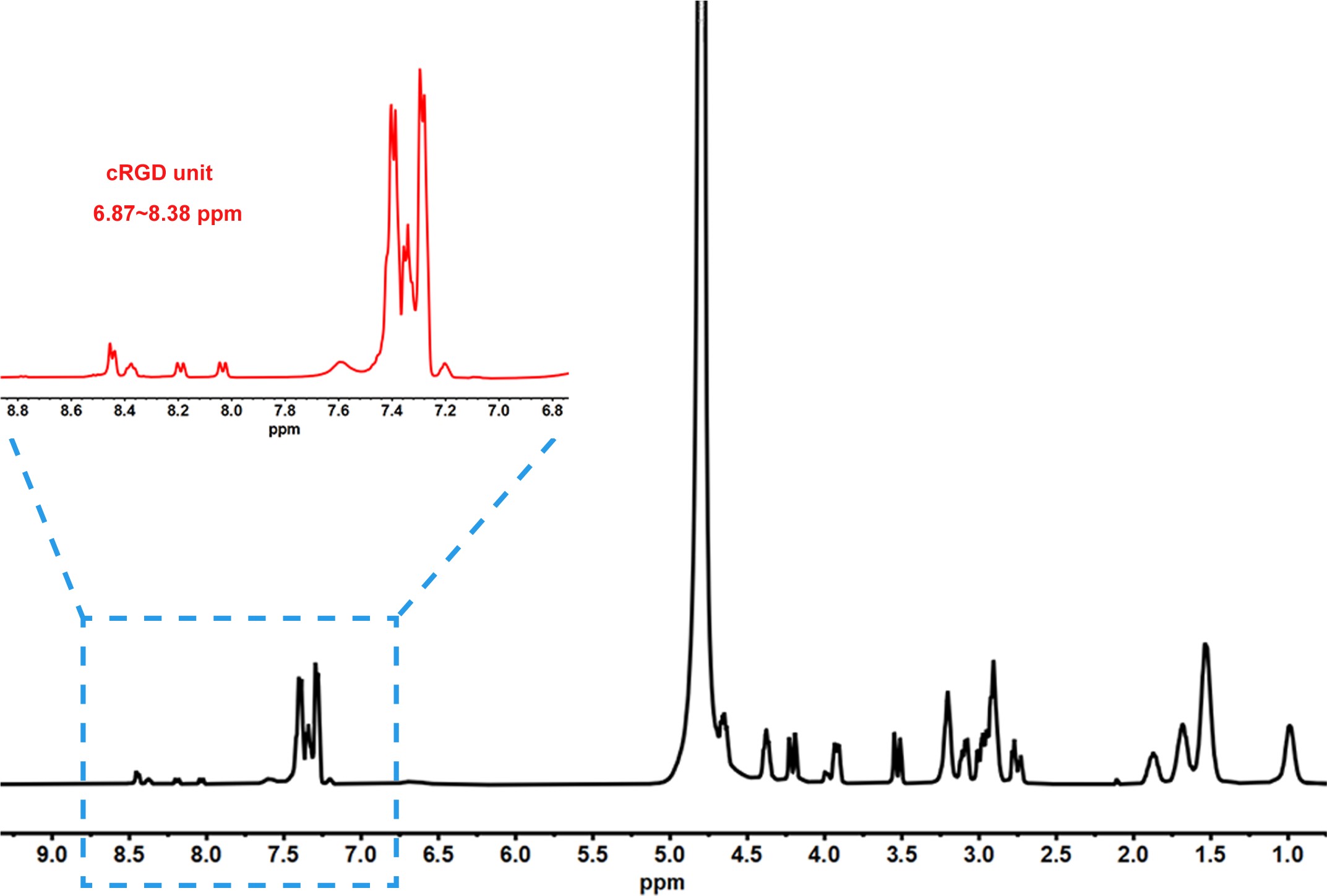

Supplement: Supplementary 1 — Figs. S1 to S19 [file research.0388.f1.zip › S5.jpg]

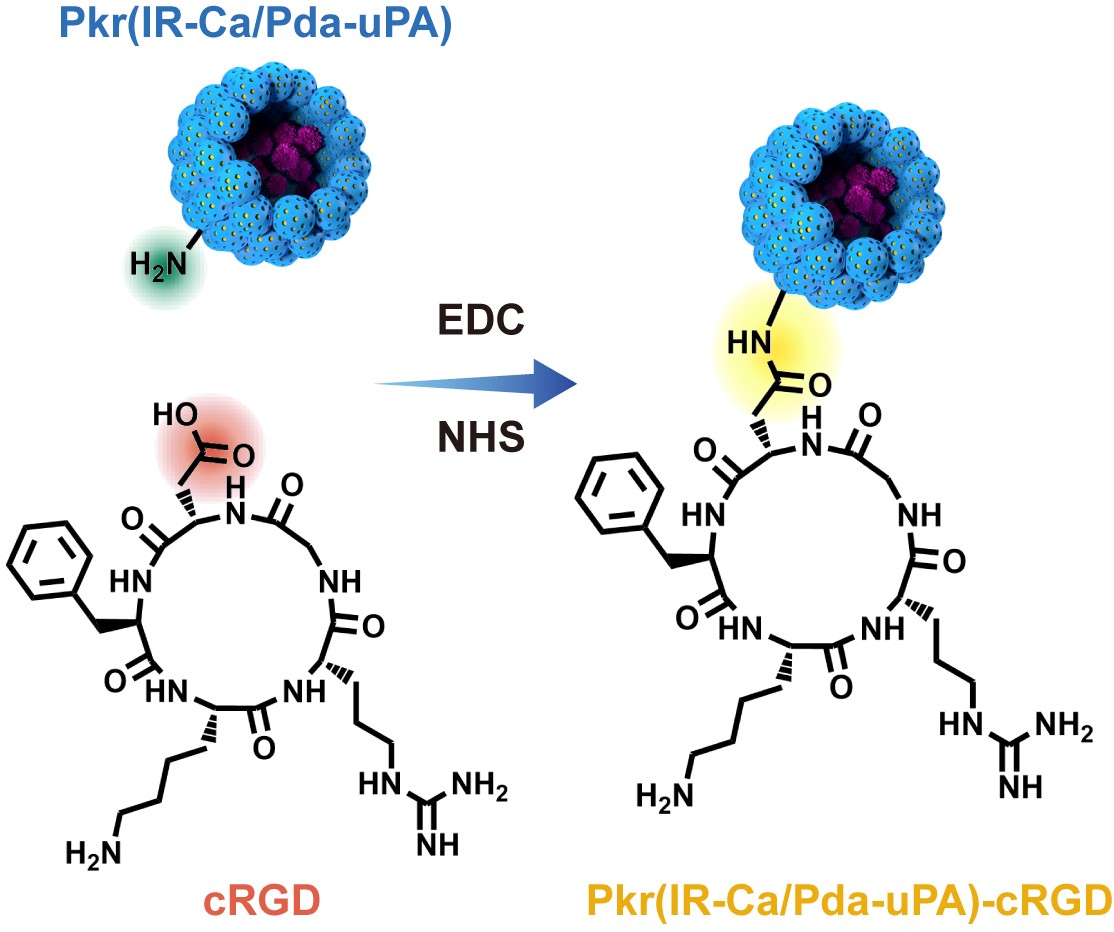

Supplement: Supplementary 1 — Figs. S1 to S19 [file research.0388.f1.zip › S4 .jpg]

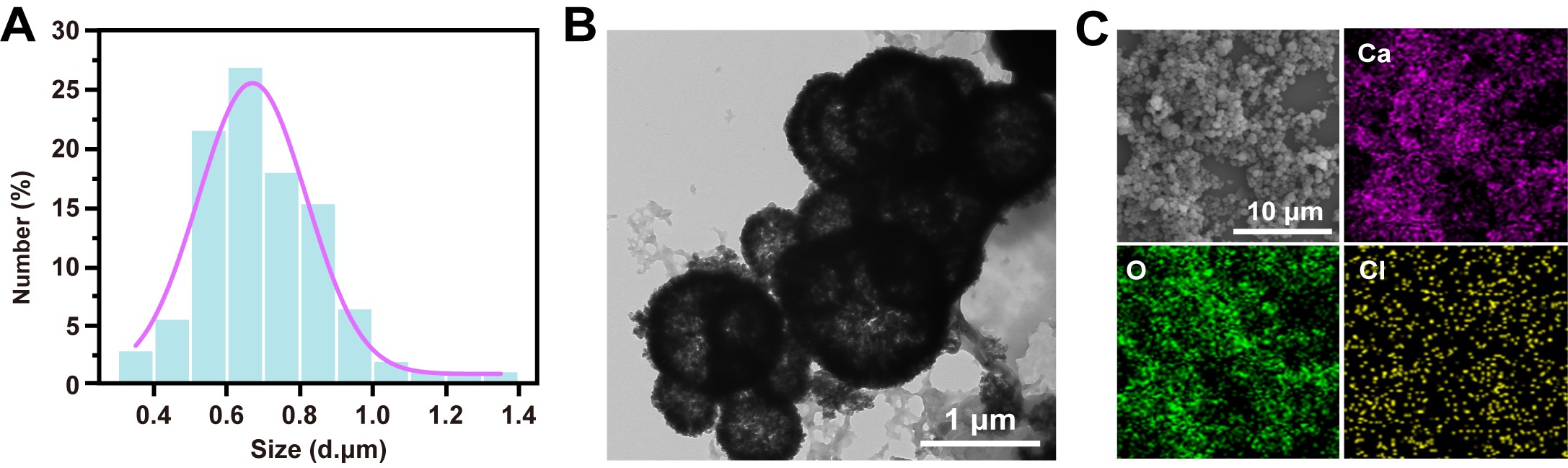

Supplement: Supplementary 1 — Figs. S1 to S19 [file research.0388.f1.zip › S3.jpg]

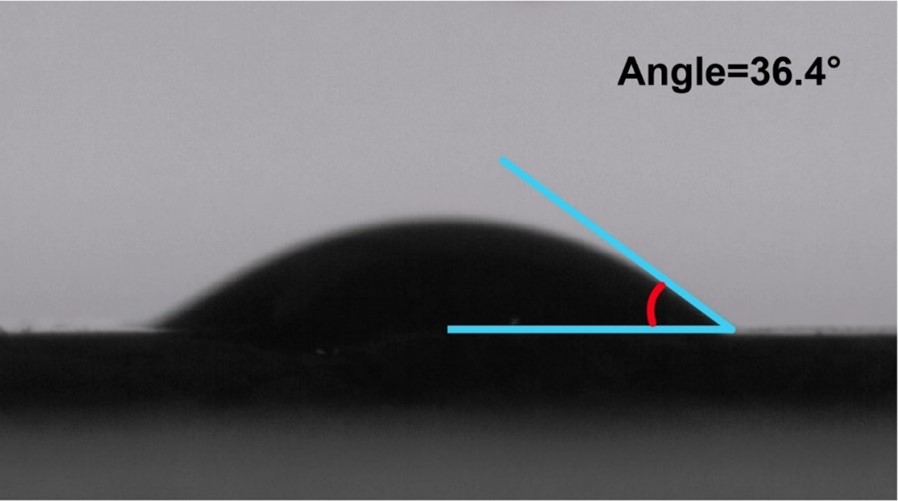

Supplement: Supplementary 1 — Figs. S1 to S19 [file research.0388.f1.zip › S2.jpg]

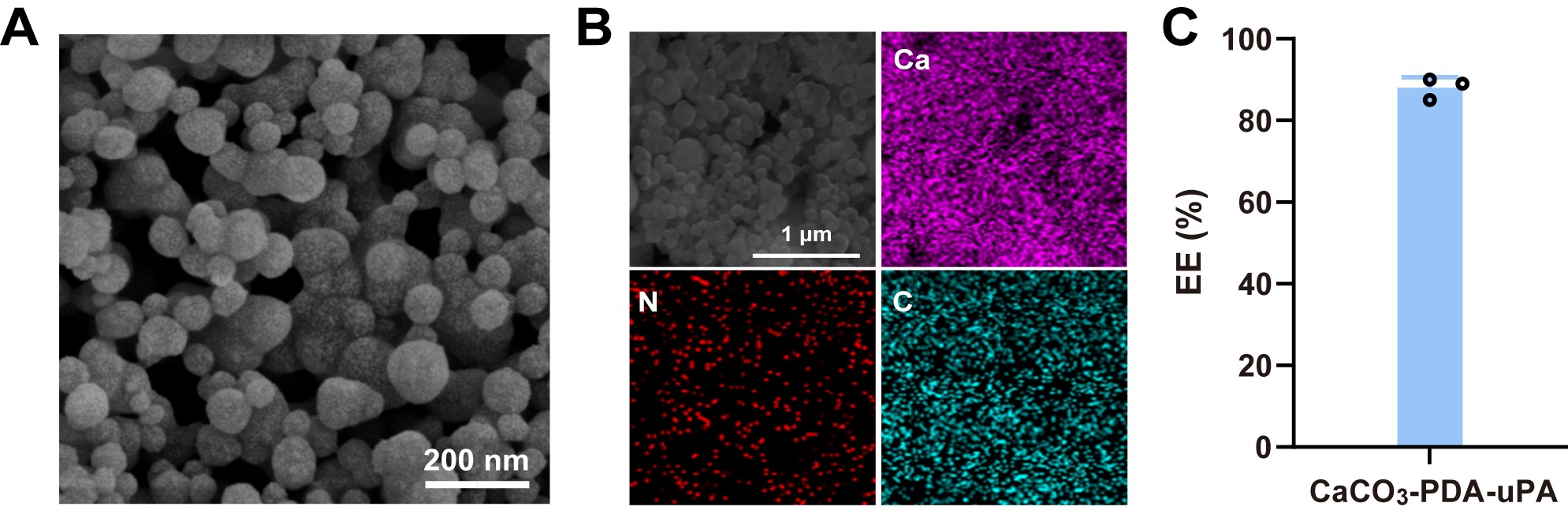

Supplement: Supplementary 1 — Figs. S1 to S19 [file research.0388.f1.zip › S1.jpg]
